# A comprehensive analysis of the animal carcinogenicity data for glyphosate from chronic exposure rodent carcinogenicity studies

**DOI:** 10.1186/s12940-020-00574-1

**Published:** 2020-02-12

**Authors:** Christopher J. Portier

**Affiliations:** 1 Rollins School of Public Health, Emory University, Atlanta, GA USA; 2 Department of Toxicogenomics, Maastricht University, Maastricht, Netherlands; 3 CJP Consulting, Seattle, Washington USA

**Keywords:** Glyphosate, Cancer, Animal carcinogenicity studies, Trend test, Historical controls

## Abstract

Since the introduction of glyphosate-tolerant genetically-modified plants, the global use of glyphosate has increased dramatically making it the most widely used pesticide on the planet. There is considerable controversy concerning the carcinogenicity of glyphosate with scientists and regulatory authorities involved in the review of glyphosate having markedly different opinions. One key aspect of these opinions is the degree to which glyphosate causes cancer in laboratory animals after lifetime exposure. In this review, twenty-one chronic exposure animal carcinogenicity studies of glyphosate are identified from regulatory documents and reviews; 13 studies are of sufficient quality and detail to be reanalyzed in this review using trend tests, historical control tests and pooled analyses. The analyses identify 37 significant tumor findings in these studies and demonstrate consistency across studies in the same sex/species/strain for many of these tumors. Considering analyses of the individual studies, the consistency of the data across studies, the pooled analyses, the historical control data, non-neoplastic lesions, mechanistic evidence and the associated scientific literature, the tumor increases seen in this review are categorized as to the strength of the evidence that glyphosate causes these cancers. The strongest evidence shows that glyphosate causes hemangiosarcomas, kidney tumors and malignant lymphomas in male CD-1 mice, hemangiomas and malignant lymphomas in female CD-1 mice, hemangiomas in female Swiss albino mice, kidney adenomas, liver adenomas, skin keratoacanthomas and skin basal cell tumors in male Sprague-Dawley rats, adrenal cortical carcinomas in female Sprague-Dawley rats and hepatocellular adenomas and skin keratocanthomas in male Wistar rats.

## Background

Glyphosate acid (CAS # 1071-81-6) is a colorless, odorless, crystalline solid. Glyphosate is the term used to describe the salt that is formulated by combining the deprotonated glyphosate acid and a cation (isopropylamine, ammonium, or sodium). Glyphosate was first synthesized in 1950 as a pharmaceutical compound but no pharmaceutical applications were identified. Glyphosate was reformulated in 1970 and tested for its herbicidal activity and was patented for use by Monsanto. The patent has since expired and now glyphosate is produced worldwide by numerous manufacturers [1]. According to the International Agency for Research on Cancer [2], glyphosate is registered in over 130 countries as of 2010. Since the introduction of genetically engineered glyphosate-tolerant crops in 1996, the global use of glyphosate has increased 15-fold making it the most widely used pesticide worldwide [3].

Most countries require a two-year rodent carcinogenicity study (cancer bioassay) be completed and the results reported to the proper authority in order to register a pesticide for use. There have been multiple cancer bioassays conducted to determine if glyphosate is potentially carcinogenic in humans. These have been reviewed by numerous regulatory agencies including the European Food Safety Authority (EFSA) [4], the European Chemicals Agency (EChA) [5], and the US Environmental Protection Agency (EPA) [6]. All of these agencies have concluded that the animal carcinogenicity data do not support a link between glyphosate and cancer. The carcinogenicity of glyphosate was also reviewed by the International Agency for Research on Cancer (IARC) [2] who found that the animal carcinogenicity data was sufficient to establish a causal link between exposure to glyphosate and cancer incidence in animals. The data have also been reviewed by the Joint Meeting of Pesticide Residues (JMPR) [7] concluding “*that glyphosate is not carcinogenic in rats but could not exclude the possibility that it is carcinogenic in mice at very high doses*.”

There is considerable controversy over the interpretation of these cancer bioassays. Numerous reasons have been put forth to explain the differences between IARC and the regulatory agencies on the carcinogenicity of glyphosate in rodents. These differences will be discussed at the end of this report.

This report considers the adequacy of the studies for addressing the carcinogenicity of glyphosate and, where data is available, reanalyzes these data to identify significant increases in tumors in these data sets and compares the results across studies.

## Main text

### Materials and methods

#### Animal carcinogenicity data

The animal carcinogenicity data derives from multiple sources including the published literature, the EPA review [6], the Addendum to the EFSA review prepared by the German Institute for Risk Analysis [8], the JMPR review [7], Additional file 1 from a review of the carcinogenicity of glyphosate by a panel of scientists on behalf of industry [9], and the full laboratory reports (with redactions) for some of these studies following a recent court decision [10] (usually these full laboratory reports are not available to the public). In some cases, only limited data is reported for a given study making comparisons to other studies difficult. Only data from the core lifetime studies are included in the evaluation; data from interim sacrifices are not included.

In total, there are 13 chronic exposure animal toxicology and carcinogenicity studies of glyphosate in rats and 8 in mice (Tables 1 and 2). The full descriptions of most studies are available in either the published document in the literature, the regulatory reports, or, where available, the full laboratory reports. Table 1 lists the 13 chronic exposure toxicity and carcinogenicity studies considered acceptable for this evaluation and provides a brief description of the species, strain, exposure levels, group sizes, chemical purity and comments on survival and weight changes seen in the study. Twelve of these studies were conducted under the appropriate regulatory guidelines at the time they were conducted. A more complete description for each of these studies including the laboratory conducting the study, the substrain of the animal used (if given), a description of pathology protocols used, a list of tissues evaluated and a complete list of all tumors analyzed in this reanalysis is provided in the Additional file 1. Table 2 identifies 8 chronic exposure toxicity and carcinogenicity studies that are not included in this evaluation and the reasons for their exclusion such as falsified data, lack of tumor data, or chemical purity.

**Table 1:** Long-term chronic dietary exposure toxicity and carcinogenicity studies of glyphosate analyzed in this evaluation. Additional information on these studies is available in the Additional file 1

Study Reference	Duration (months)	Strain		Dietary exposure dose levels (mg/kg/day)	Animals per Group	Purity (%)	Comments on survival and weight
		Mouse	Rat				
A: Knezevich and Hogan (1983) [11]	24	CD-1		M: 0, 157, 814, 4841 F: 0, 190, 955, 5874	50	99.8	No survival differences, slight weight reduction in high dose (M)
B: Atkinson et al. (1993) [12]	24	CD-1		M: 0, 98, 297, 988 F: 0, 102, 298, 1000	50	> 97.0	No survival differences, no weight differences
C: Sugimoto (1997) [13]	18	CD-1		M: 0, 165, 838.1, 4348 F: 0, 153.2, 786.8, 4116	50	94.6–95.7	No survival differences, slight weight reduction in mid (F) & high dose (M + F)
D: Wood et al. (2009) [14]	18	CD-1		M: 0, 71.4, 234.2, 810 F: 0, 97.9, 299.5, 1081.2	51	95.7	No survival differences, no weight differences
E: Takahashi (1999a) [15]	18	CD-1		M: 0, 167.6, 685, 7470 F: 0, 93.2, 909, 8690	50	97.5	Reduced survival high dose (M), slight weight reduction in mid (M) & high dose (M + F). This study was only mentioned by JMPR [7] and provides limited tumor data.
F: Kumar (2001) [16]	18	S-A^a^		M: 0, 85.5, 285.2, 1077.4 F: 0, 104.5, 348.6, 1381.9	50	> 95.0	No survival differences, no weight differences
G: Lankas (1981) [17]	26		SD^b^	M: 0, 3.05, 10.3, 31.49 F: 0, 3.37, 11.22, 34.02	50	98.7	No survival differences, no weight differences
H: Stout and Ruecker (1990) [18]	24		SD^b^	M: 89, 362, 940 F: 0, 113, 457, 1183	50	98.7	No survival differences, slight weight reduction in high dose (F)
I: Atkinson (1993) [19]	24		SD^b^	M: 0, 11, 112, 320, 1147 F: 0, 12, 109, 347, 1134	50	98.9	No survival differences, slight weight reduction in high dose (M + F)
J: Enemoto (1997) [20]	24		SD^b^	M: 0, 104, 354, 1127 F: 0, 115, 393, 1247	50	95.7	Reduced survival high dose (M), slight weight reduction in high dose (M + F)
K: Suresh (1996) [21]	24		W^c^	M: 0, 6.3, 59.4, 595.2 F: 0, 8.6, 88.5, 886	50	96.8	No survival differences, no weight differences
L: Brammer (2001) [22]	24		W^c^	M: 0, 121, 361, 1214 F: 0, 145, 437, 1498	53	97.6	High-dose survived longer (M), reduced weight highest dose (M + F)
M: Wood et al. (2009) [23]	24		W^c^	M: 0, 165, 838.1, 4348 F: 0, 153.2, 786.8, 4116	51	94.7–97.6	No survival differences, no weight differences

^a^Swiss Albino mouse; ^b^Sprague-Dawley rat; ^c^Wistar rat

**Table 2:** Long-term chronic dietary exposure toxicity and carcinogenicity studies of glyphosate excluded from this evaluation

Study Reference	Duration (months)	Strain		Dietary exposure dose levels (mg/kg/day)	Animals per Group	Purity (%)	Reason for exclusion
		Mouse	Rat				
Reyna and Gordon (1973) [24]	18	SWM^a^		M: 0, 17,50 F: 0, 17,50	50	NP^b^	EPA [25] concluded this study was likely falsified
Pavkov and Turner (1987) [26]	24	CD-1		M: 0, 11.7, 118, 991 F: 0, 11.7, 118, 991	50	56.2	EPA [6] lists this study as completely negative for tumors but provides no tumor data. No tumor data is available for this study and the purity is low.
Reyna and Gordon (1974) [27]	24		SD^c^	Not available	70	13.8	EPA [25] concluded this study was likely falsified
Burnett et al. (1979) [28]	24		SD^c^	M: 0, 3.10.30 F: 0, 3,10,30	90	NP^b^	EPA initially reported this as a glyphosate study [29] but later removed it because it is a study of a contaminant of glyphosate [6].
Pavkov and Wyand (1987) [30]	24		SD^c^	M: 0, 4.2, 21.2, 41.8 F: 0, 5.4, 27, 55.7	80–90	56.2	EPA [6] lists this study as completely negative for tumors but provides very limited tumor data [31]. No tumor data is available for this study and the purity is low.
Excel (1997) [32]	24		SD^c^	M: 0, 150, 780, 1290 F: 0, 210, 1060, 1740	51	NP^b^	No tumor data available, regulatory agencies had concerns about the quality of the study and purity of the material being studied
Takahashi (1999b) [33]	24		F^d^	M: 0, 25, 201, 1750 F: 0, 29.7, 239, 2000	50	NP^b^	This study is only mentioned by JMPR [7] and showed body weight changes at the highest exposure which probably exceeded the MTD. No tumor data were provided although JMPR concluded there is no increased carcinogenicity.
Chruscielska (2000) [34]	24		W^e^	M: 1.9, 5.9, 17 F: 0, 2.2, 6.5, 19	85	GBH^f^	Uncertainty in the material used in the study and poor reporting in the study. Note: this study is in drinking water

^a^Swiss white mouse; ^b^Purity not provided; ^c^Sprague-Dawley rat; ^d^Fischer F344 rats; ^e^Wistar rats; ^f^glyphosate-based herbicide (13.8% solution, probably Perzocyd according to Greim et al. [9])

For 12 of these studies, the full study report is available. For study E (Takahashi [15]), a full study report is not available. JMPR [7] provided the only review of this study and only reported on kidney tumors in males and malignant lymphomas in females. This study is included in this review for only kidney tumors in males and malignant lymphomas in females.

Two additional chronic exposure studies of glyphosate formulations are included in this review as additional support for the carcinogenicity of glyphosate. These studies are not reanalyzed for this evaluation; the evaluations of the original authors are described in the Results section.

George et al. [35] exposed groups of 20 male Swiss Albino mice to a glyphosate formulation (Roundup Original, 360 g/L glyphosate) at a dose of 25 mg/kg (glyphosate equivalent dose) topically three times per week, topically once followed one week later by 12-o-tetradecanoylphorbol-13-acetate (TPA) three times per week, topically three times per week for three weeks followed one week later by TPA three times per week, or a single topical application of 7,12-dimethyl-benz[a]anthracene (DMBA) followed one week later by topical application of glyphosate three times per week for a total period of 32 weeks. Appropriate untreated, DMBA-treated, and TPA-treated controls were included.

Seralini, G. E., et al. [36] conducted a 24-month chronic toxicity study of Roundup (GT Plus, 450 g glyphosate/L, EU approval 2,020,448) in groups of 10 male and female Sprague-Dawley rats with drinking-water exposures of 0, 1.11•10^− 8^, 0.09, and 0.5% Roundup (males and females). This study noted an increase in mammary tumors. However, given the small sample sizes employed and the availability of more detailed studies, this study will be included in this review only as supporting information.

#### Data analysis

Individual tumor counts for the individual studies are reanalyzed using the exact form of the Cochran-Armitage (C-A) linear trend test in proportions [37]. Reanalyses are conducted on all primary tumors where there are at least 3 tumors in all of the animals in a sex/species/strain combination (regardless of dosing). In addition, any tumor where a positive finding (*p* ≤ 0.05, one-sided C-A trend test) is seen in at least one study is also evaluated, regardless of number of animals with the tumor, in all studies of the same sex/species/strain. When adenomas and carcinomas are seen in the same tissue, a combined analysis of adenomas and carcinomas is also conducted. The minimum of three tumors is used since the exact version of the C-A test cannot detect tumors in studies of this size with less than at least 3 tumors. Additional file 2: Tables S1–S13 provide the tumor count data for all tumors with a significant trend test (*p* ≤ 0.05) in at least one study of the same sex/species/strain along with the doses used (mg/kg/day) and the number of animals examined microscopically in each group. Pairwise comparisons between individual exposed groups and control are conducted using Fisher’s exact test [37] and are provided for comparison with other reviews.

The C-A trend test belongs to the general class of logistic regression models [37]. To evaluate the consistency of a tumor finding across multiple studies using the same sex-species-strain combinations, logistic regression with individual background responses and dose trends are fit to the pooled data using maximum likelihood estimation. In mathematical terms, the regression model being used is:

1 $$ p=\frac{e^{\alpha_i+\beta \cdot dose}}{1+{e}^{\alpha_i+\beta \cdot dose}} $$

where *p* is the probability of having a tumor, *α*_*i*_ is a parameter associated with the background tumor response (dose = 0) for study *i* and *β* is a parameter associated with a change in the tumor response per unit dose (slope). A common positive trend is seen in the pooled analysis when the null hypothesis that the slope is 0 (H_0_: *β* =0) is rejected (statistical *p*-value ≤0.05 using a likelihood-ratio test) in favor of the alternative that the slope is greater than 0 (H_A_: *β* > 0). The heterogeneity of slopes (all studies have different slopes vs all studies have a common slope) is tested using the model:

2 $$ p=\frac{e^{\alpha_i+{\beta}_i\cdot dose}}{1+{e}^{\alpha_i+{\beta}_i\cdot dose}} $$

where *p* and *α*_*i*_ are as in equation (1) and *β*_*i*_ is a parameter associated with the slope for study *i*. Heterogeniety is seen in the pooled analysis when the null hypothesis that the slopes are equal (H_0_: *β*_1_ = *β*_2_ = *β*_3_ =…) is rejected (statistical *p*-value ≤0.05 using a likelihood-ratio test) in favor of the alternative that at least one of the slopes is different.

For CD-1 mice, there are studies of 18 months (3) and 24 months (2) so analyses are conducted separately for 18 month studies and 24 month studies and then a combined analysis is performed. In SD rats, one study had 26 months of exposure and the remaining 3 had 24 months of exposure so similar grouped analyses are conducted. Only the combined analysis over all study durations is provided in Tables 3, 4 and 5; the sub-analyses by study duration are discussed in the text.

**Table 3:** *P*-values for the Cochran-Armitage trend test and pooled logistic regression analysis for tumors with at least one significant trend test (*p* ≤ 0.05) or Fisher’s exact test (*p* ≤ 0.05) in male and female CD-1 mice

Tumor	Individual study *p*-values for trend^a^					Common Trend	Heterogeneity Test
Males	A	B	C	D	E		
Kidney Adenomas	0.442 (0.138)^d^	0.938	0.062 (**0.009**)^d^	---^b^	**0.019**	**0.006**	0.268
Kidney Carcinomas	0.063 **(< 0.001**)^d^	0.938	---^b^	---^b^	0.250	**0.031**	0.546
Kidney Adenomas and Carcinomas	0.065 (**0.008**)^d^	0.981	0.062 (**0.009**)^d^	---^b^	**0.005**	**< 0.001**	0.106
Malignant Lymphomas	0.754	0.087	**0.016**	**0.007**	ND^c^	0.093	0.007
Hemangiosarcomas	0.505	**0.004**	0.062 (**0.005**)^d^	---^b^	ND^c^	**0.033**	0.007
Alveolar-Bronchiolar Adenomas	0.294	0.231	0.513	0.924	ND^c^	0.384	0.409
Alveolar-Bronchiolar Carcinomas	0.918	0.456	0.148	**0.028**	ND^c^	0.407	0.083
Alveolar-Bronchiolar Adenomas and Carcinomas	0.576	0.231	0.294	0.336	ND^c^	0.346	0.826
Females	A	B	C	D	E		
Hemangiomas	0.631	---^b^	**0.002**	0.438	ND^c^	**0.031**	0.155
Harderian Gland Adenomas	0.877	ND^c^	**0.040**	0.155	ND^c^	0.155	0.052
Harderian Gland Carcinomas	---^b^	ND^c^	---^b^	1.000	ND^c^	0.500	1.00
Harderian Gland Adenomas and Carcinomas	0.877	ND^c^	**0.040**	0.372	ND^c^	0.184	0.110
Alveolar-Bronchiolar Adenomas	0.999	0.144	0.800	0.656	ND^c^	0.996	0.211
Alveolar-Bronchiolar Carcinomas	0.183	0.110	0.623	0.601	ND^c^	0.268	0.544
Alveolar-Bronchiolar Adenomas and Carcinomas	0.985	**0.048**	0.842	0.688	ND^c^	0.982	0.241
Malignant Lymphomas	0.070^e^	0.484	0.294	0.353	**0.050**	**0.012**	0.995

^a^ – Study A is Knezevich and Hogan [11] (Additional file 2: Table S1), Study B is Atkinson et al. [12] (Additional file 2: Table S2), Study C is Sugimoto [13] (Additional file 2: Table S3), Study D is Wood [14] (Additional file 2: Table S4), Study E is Takahashi [15] (Additional file 2: Table S5); ^b^ – three dashes “---” indicates all tumor counts are zero; ^c^ – ND indicates there is no data available for this tumor in this study; ^d^ – using historical control data (see text for details) and Tarone’s test; ^e^ – Spleen composite lymphosarcomas (malignant lymphomas) are also significantly increased in female mice in this study (see Additional file 2: Table S1)

**Table 4:** *P*-values for the Cochran-Armitage trend test and pooled logistic regression analysis for tumors with at least one significant trend test or Fisher’s exact test (*p* ≤ 0.05) in male and female Sprague-Dawley rats

Tumor	Individual study *p*-values for trend^a^				Common Trend	Heterogeneity Test
Males	G	H	I	J		
Testicular Interstitial Cell Tumors	**0.009**	0.296	0.580	0.594	0.461	0.105
Pancreas Islet Cell Adenomas	0.512	0.147 (**0.007**)^c^	0.974	0.859	0.849	0.143
Pancreas Islet Cell Carcinomas	0.251	1.000	–	0.500	0.731	0.166
Pancreas Islet Cell Adenomas or Carcinomas	0.316	0.206	0.974	0.844	0.875	0.185
Thyroid C-cell Adenomas	0.743	0.089	0.278	0.631	0.210	0.532
Thyroid C-cell Carcinomas	0.505	0.442	0.495	0.565	0.322	0.898
Thyroid C-cell Adenomas and Carcinomas	0.748	0.097	0.197	0.642	0.175	0.526
Thyroid Follicular-cell Adenomas	0.122	0.408	0.067	0.966	0.464	0.055
Thyroid Follicular-cell Carcinomas	---^b^	0.255	0.443	1.000	0.448	0.137
Thyroid Follicular-cell Adenoma and Carcinoma	0.122	0.232	0.099	0.986	0.446	0.031
Hepatocellular Adenomas	0.471	**0.015**	0.325	0.500	**0.029**	0.664
Hepatocellular Carcinomas	0.062	0.637	0.760	0.642	0.803	0.269
Hepatocellular Adenomas and Carcinomas	0.173	**0.050**	0.480	0.690	0.144	0.428
Kidney Adenomas	0.938	0.813	1.000	**0.004**	**0.039**	0.002
Skin Keratoacanthomas	---^b^	**0.042**	**0.047**	**0.029**	**< 0.001**	0.998
Skin Basal Cell Tumors	0.251	0.249	1.000	**0.004**	**< 0.001**	0.009
Females	G	H	I	J		
Thyroid C-cell Adenomas	0.679	**0.049**	0.207	0.912	0.287	0.150
Thyroid C-cell Carcinomas	**0.003 (< 0.001)**^**c**^	0.500	---^b^	---^b^	0.385	0.041
Thyroid C-cell Adenomas and Carcinomas	0.072 **(0.037)**^**c**^	0.052	0.207	0.912	0.275	0.071
Adrenal Cortical Adenoma	0.851	0.603	---^b^	0.626	0.713	0.750
Adrenal Cortical Carcinoma	0.386	**0.015**	0.493	---^b^	**0.031**	0.199
Adrenal Cortical Adenoma and Carcinoma	0.801	0.090	0.493	0.626	0.195	0.520

^a^ – Study G is Lankas [17] (Additional file 2: Table S7), Study H is Stout and Ruecker [18] (Additional file 2: Table S8), Study I is Atkinson et al. [12] (Additional file 2: Table S9) and Study J is Enemoto [20] (Additional file 2: Table S10); ^b^ – three dashes “---” indicates all tumor counts are zero; ^c^ – using historical control data (see text for details) and Tarone’s test

**Table 5:** *P*-values for the Cochran-Armitage trend test and pooled logistic regression analysis for tumors with at least one significant trend test or Fisher’s exact test (*p* ≤ 0.05) in male and female Wistar rats

Tumor	Individual study *p*-values for trend^a^			Common Trend	Homogeneity Test
Males	K	L	M		
Hepatocellular Adenomas	0.391	**0.008**	0.418	**0.048**	0.156
Hepatocellular Carcinomas	0.418	---^b^	1.000	0.492	0.242
Hepatocellular Adenomas and Carcinomas	0.286	**0.008**	0.610	**0.029**	0.194
Pituitary Adenomas	0.376	0.277	**0.045**	0.057	0.664
Pituitary Carcinomas	0.692	---^b^	1.000	0.771	0.956
Pituitary Adenomas and Carcinomas	0.454	0.277	0.059	0.073	0.700
Skin Keratoacanthomas	---^b^	0.387	**0.030**	**0.032**	0.823
Adrenal Pheochromocytomas	**0.048**	0.721	0.306	0.273	0.210
Females	K	L	M		
Mammary Gland Adenomas	0.539	0.941	0.062	0.448	0.015
Mammary Gland Adenocarcinomas	1.000	0.271	**0.042**	0.071	0.008
Mammary Gland Adenomas and Adenocarcinomas	0.729	0.590	**0.007**	0.113	0.064
Pituitary Adenomas	0.967	0.261	**0.014**	0.105	0.023
Pituitary Carcinomas	1.000	–	0.750	0.748	0.491
Pituitary Adenomas and Carcinomas	0.976	0.261	**0.017**	0.129	0.019

^a^ – Study J is Suresh [21] (Additional file 2: Table S11), Study K is Brammer [22] (Additional file 2: Table S12), and Study L is Wood et al. [14] (Additional file 2: Table S13); ^b^ – three dashes “---” indicates all tumor counts are zero

The same methods of analysis are used to evaluate the incidence of non-cancerous toxicity in tissues where positive cancer findings are seen. These findings are discussed in the text but not shown in the tables.

In some cases, tumors that rarely (< 1% in untreated animals) appear in laboratory animals can be increased but do not show statistical significance. Most guidelines call for the use of historical control data to evaluate these cases to assess the significance of the findings [38–40]. For these evaluations, the test proposed by Tarone [41] is used with an appropriate historical control group as discussed in the text.

All analyses were done using MATLAB, version R2017b.

## Conclusions

### Results

Thirteen chronic exposure animal carcinogenicity studies are reviewed and reanalyzed for this evaluation. The summary of all tumor findings with a Cochran-Armitage (C-A) trend test (one-sided) of *p* ≤ 0.05 in at least one study (by sex/species/strain) from the reanalysis of these studies are provided in Tables 3, 4 and 5 (columns under the heading “Individual study p-values for trend”). In addition, the p-values for trend (under the heading “Common Trend”) and heterogeneity (under the heading “Heterogeneity Test”) from the analysis of the pooled data are also provided in Tables 3, 4 and 5. The individual tumor counts for each individual study are shown in Additional file 2: Tables S1–S13. In addition, a few tumors where there is a significant (*p* ≤ 0.05) pairwise comparison by Fishers exact test in at least one study but no significant trend tests are also summarized in Tables 3, 4 and 5; this is for comparison with regulatory reviews that generally used only pairwise comparisons.

The purpose of this analysis is to understand the tumorogenicity of glyphosate across all studies and not one study at a time. Thus, rather than presenting the results of each study separately, this review focuses on the tumors that are seen as positive in any one study and compares the findings across all studies of the same tumor in the same sex/species/strain combination.

#### Reanalysis of the data from CD-1 Mice

Table 3 summarizes the significant results seen from five studies conducted in CD-1 mice [11–15]. For a complete list of all the tumors evaluated, see the Additional file 1. For simplicity, these studies will be referred to as studies A-E as noted in Table 1. Studies A and B are 24-month studies and studies C, D and E are 18-month studies. There are a total of 12 statistically significant tumor findings (*p* ≤ 0.05) against the concurrent controls in these studies. In addition, there are 5 significant increases in tumors seen for rare tumors using historical controls.

Significant trends for kidney adenomas (*p* = 0.019) and adenomas and carcinomas combined (*p* = 0.005) are seen in male mice in study E, marginal trends are seen in study A (*p* = 0.065) and study C (0.062) for combined adenomas and carcinomas with no increase in the remaining two studies. Kidney tumors are rare in CD-1 mice and it would be appropriate to compare the marginal responses against historical controls. Using historical control data for kidney tumors from the EPA archives [42] on study A results in no significant association with adenomas (*p* = 0.138) but significant increases in carcinomas (*p* < 0.001) and adenomas and carcinomas combined (*p* = 0.008) by Tarone’s test. Using historical controls from 1990 to 1995 from the literature [43] results in a significant trend (*p* = 0.009) for kidney adenomas in Study C. The pooled analysis of the data shows a significant common trend for adenomas, carcinomas and the combined tumors with no indication of heterogeneity. Because of toxicity in the highest dose of study E, a second pooled analysis is done dropping this dose and yields a significant increase for adenomas (*p* = 0.038) and carcinomas and adenomas combined (*p* = 0.011) and a marginal increase for carcinomas (*p* = 0.077) with no heterogeneity (not shown). Data on the incidence of kidney toxicity in these studies is also reanalyzed. Study A has a significant increase in chronic interstitial nephritis (*p* = 0.004) and a non-significant increase in thickening of the glomerular and/or tubular basal membranes (*p* = 0.148) with a significant pairwise increase at the mid-dose (*p* = 0.036). Study B has an increase in tubular dilatation (*p* = 0.026) but no change in tubular hypertrophy (*p* = 0.642) or focal tubular atrophy (*p* = 0.248). Study C has no change in tubular dilatation (*p* = 0.913) but does show an increase in tubular atrophy (*p* = 0.017) and tubular vacuolation (*p* = 0.015). Study D has no changes in vacuolation (*p* = 0.830), dilatation (*p* = 0.831), or chronic nephropathy (*p* = 0.494). Study E has an increase in kidney tubular dilation (*p* < 0.001), tubular epithelial cell hypertrophy (*p* < 0.001), basophilic tubules (*p* = 0.009) and tubular degeneration and/or necrosis (*p* = 0.008).

Malignant lymphomas are significant in studies C (*p* = 0.016) and D (*p* = 0.007) and marginally significant in study B (*p* = 0.087) in male mice. Malignant lymphomas are not rare in these mice so no historical control analysis is conducted. The pooled analysis for a common trend is marginally significant (*p* = 0.093) and the studies are heterogeneous in slope because of the markedly different response in study A. The pooled analysis of the 18 month studies is highly significant (*p* = 0.005) but not significant for the 24 month studies (*p* = 0.686). Toxicity in tissues relating to the lymphatic system is reanalyzed. Study B shows a significant increase in thymus weight in the two highest exposure groups (*p* < 0.01 and *p* < 0.05, reported in [12]) in males and a non-significant (p not reported) increase in females. Studies B and C show a significant increase (trend test) in the number of males with enlarged mesenteric lymph nodes (*p* = 0.024 and *p* = 0.002 respectively). Study B shows enlarged spleens (*p* = 0.031) in males whereas C did not. Study C also has an increase in enlarged cervical lymph nodes (*p* = 0.046) and other lymph nodes (*p* = 0.047). Study A did not report macroscopic findings, study D has no enlarged lymphoreticular tissues and the data are not available from study E.

Hemangiosarcomas are statistically significant in study B (*p* = 0.004) and marginally significant in study C (*p* = 0.062) in male mice. Hemangiosarcomas are very rare in 18-month animals with no tumors appearing in 26 historical control data sets and moderately rare (2.1%) in 24-month studies [43]. Using the 18-month historical control data [43] results in a significant finding for study C (*p* < 0.001). The pooled analysis for a common trend is significant (*p* = 0.03) but the studies are heterogeneous in slope.

Although there is a single positive finding in the lung in male mice with a significant increase in carcinomas in study D (*p* = 0.028), all of the other analyses in the lung are not statistically significant including the pooled analyses. There are no dose-related non-neoplastic findings in the lungs of these animals.

In female mice, hemangiomas are significantly increased in study C (*p* = 0.002) and the pooled analyses is also significant (*p* = 0.031) with no evidence of heterogeneity. Study C has a 10% response at the highest dose whereas the other studies have much lower response resulting in the positive pooled association.

Harderian gland adenomas are significantly increased in study C (*p* = 0.04) but are not significant for studies A and D for adenomas, carcinomas and their combination. The pooled analyses fails to demonstrate a consistent increase. There are no non-neoplastic findings in the Harderian glands.

There is a significant increase in adenomas and carcinomas combined in the lung for female mice in study B (*p* = 0.048). None of the pooled analyses or any analyses in the remaining studies are significantly increased in the lung. There are no non-neoplastic findings in the lungs of these animals.

Finally, malignant lymphomas are significantly increased in study E (*p* = 0.050) and marginally increased in study A (*p* = 0.070) for females. The remaining studies show trends toward increasing risk with increasing exposure and when combined, the five mice studies show a significant increase in malignant lymphomas in female mice (*p* = 0.012) and no heterogeneity. The pooled analysis remains significant (p = 0.050) if the high dose group from study E is removed due to high toxicity. There are no increases in enlargement of lymphoreticular tissues in female mice in studies B, C and D and no data available for studies A and E.

#### Reanalysis of the data from Swiss albino mice

There is a single study in Swiss albino mice (study F). This study shows a significant increase in hemangiomas in female mice (*p* = 0.004) and marginal increases for malignant lymphomas in males (*p* = 0.064) and females (*p* = 0.070) and kidney adenomas in males (*p* = 0.090) (Additional file 2: Table S6). There are no kidney carcinomas in the males. There are no non-neoplastic changes in the kidney. Study F shows a significant increase in the incidence of thymus enlargement in males (*p* = 0.034) and a marginal increase in enlargement of mesenteric lymph nodes in females (*p* = 0.053) but not in males. For a complete list of all the tumors evaluated, see the Additional file 1.

#### Reanalysis of the data from SD rats

Table 4 summarizes the significant results seen from four studies conducted in SD rats [17–20]. For a complete list of all the tumors evaluated, see the Additional file 1. Study G is a 26-month study and studies H, I and J are 24-month studies. There are a total of 11 statistically significant tumor findings (*p* ≤ 0.05) against the concurrent controls in these studies and three significant finding against historical controls.

Study G showed a significant increase in testes interstitial-cell tumors (p = 0.009) but no increases in any other study and the pooled analysis for a common trend is also non-significant. There are no non-neoplastic lesions seen in the testis in studies G, H and J. Study I saw a marginal increase (*p* = 0.092) in interstitial cell hyperplasia of the testis.

Pancreas islet-cell tumors, thyroid c-cell tumors and thyroid follicular-cell adenomas and carcinomas in males are presented in Table 4. None of these studies demonstrate a significant trend in any of these tumors nor do they show a significant trend in the pooled analyses. These tumors are included here for completeness because they have been mentioned in some of the regulatory reviews of these data due to increases in at least one dose group over controls using Fisher’s exact test. Study G shows an increase in pancreatic islet cell adenomas in males at the low dose and study H shows increases in males at both the low dose and the high dose. Historical control data on pancreas islet-cell tumors in study H are provided in an EPA memo [44] and Tarone’s historical control test yields a highly significant response for this study (*p* = 0.007) with all of the treated groups showing greater tumor response than any of the controls. There are no dose-related increases in islet cell non-neoplastic findings in any of the four studies in male Sprague-Dawley rats.

Study H saw an increase in males of thyroid C-cell adenomas at the mid and high doses and an increase in adenomas and carcinomas combined at all three doses tested. However, the control response in study H for these tumors is quite low with no tumors in 50 animals whereas the historical rate of tumors in this strain of rats is 11.3% in males [45]. Reanalyzing data on non-neoplastic toxicity, Study I has a significant increase in focal C-cell hyperplasia (*p* = 0.048) and no other studies have significant increases in C-cell hyperplasia.

Study I shows a marginally significant trend in males of thyroid follicular cell adenomas (*p* = 0.067) and adenomas and carcinomas combined (*p* = 0.099). No non-neoplastic endpoints show dose-related changes for thyroid follicular cells in any study.

Hepatocellular adenomas (*p* = 0.015) and adenomas and carcinomas combined (*p* = 0.050) are increased in males in study I but not in any of the other studies. The increases in adenomas remained significant (*p* = 0.029) in the pooled analysis since most studies showed a very slight increase in these tumors, but the pooled analysis for a common trend in adenomas and carcinomas is not significant (*p* = 0.144). After reanalysis of these studies for non-neoplastic toxicity, study G shows a significant increase in basophilic foci (p = 0.029), study H did not report on these and studies I and J show non-significant trends with the pooled analysis for a common trend not significant (*p* = 0.358). Study G has an increase in clear-cell foci (*p* = 0.033), study I has a marginal increase in clear-cell foci (*p* = 0.057) and study J is non-significant with the pooled analysis showing a marginally significant trend (*p* = 0.073).

Kidney adenomas are increased in males (*p* = 0.004) in study J but not in any other study. The pooled analysis for a common trend is significant (*p* = 0.039) with significant heterogeneity because of the high response in study J and the generally low response in the remaining three studies. The only non-neoplastic pathology in the kidney is an increase in lymphocytic infiltration (*p* = 0.037) in study G.

No skin keratoacanthomas are seen in males in study F, but these tumors are significantly increased in the other three studies (*p* = 0.042, 0.047 and 0.029) and are highly significant in the pooled analysis for a common trend (*p* < 0.001) with no apparent heterogeneity. After reanalysis of non-neoplastic toxicity, focal hyperkeratosis is increased in both sexes (*p* ≤ 0.001 – M; *p* = 0.015 – F) in study J and shows a significant decrease in study I in males (*p* = 0.004).

Skin basal cell tumors in males are significantly increased in study J (p = 0.004) and in the pooled analysis for a common trend (*p* < 0.001) but not in any of the other three studies. The pooled analysis demonstrates significant heterogeneity (*p* = 0.009), driven by the responses at lower doses in studies G and H.

In females, thyroid C-cell adenomas are significantly increased in study H (*p* = 0.049), carcinomas are significantly increased in study G (*p* = 0.003) and adenomas and carcinomas combined are marginally significantly increased in studies G (*p* = 0.072) and H (*p* = 0.052). The authors of study G provided historical control data from 9 control groups for carcinomas and adenomas and carcinomas combined for these tumors; Tarone’s test yielded *p* < 0.001 for the carcinomas and *p* = 0.037 for the combined tumors. None of the pooled analyses are statistically significant. There are no non-neoplastic changes in thyroid C-cells in females in these studies.

Adrenal cortical carcinomas are increased in females in study H (*p* = 0.015) and adenomas and carcinomas are marginally increased (*p* = 0.090) in that same study. The pooled analysis for a common trend of the cortical carcinomas is significantly increased (*p* = 0.031) with little indication of heterogeneity, but the pooled analysis of the combined adenomas and carcinomas is not significantly increased. After reanalysis of non-neoplastic toxicity, focal cortical hypertrophy shows a dose-related significant increase in studies G (*p* = 0.048) and I (*p* = 0.027), study H did not report hypertrophy independent of hyperplasia (the combined counts showed no increased dose-response), and study J did not report hypertrophy. There are no other dose-related increases in injury to adrenal cortical tissue in any of the studies.

#### Reanalysis of the data from Wistar rats

Table 5 summarizes the significant results seen from three studies conducted in Wistar rats [21–23]. For a complete list of all the tumors evaluated, see the Additional file 1. All three studies are 24-month studies. There are a total of 9 statistically significant tumor findings (*p* ≤ 0.05) against the concurrent controls in these studies.

Hepatocellular adenomas (*p* = 0.008) and combined adenomas and carcinomas (*p* = 0.008) in males are increased in study L but not in any other study (note, there are no carcinomas seen in this study so these analyses are identical). The pooled analyses for a common trend shows an increase for adenomas (*p* = 0.048), no increase in carcinomas (0.492) and an increase in combined adenomas and carcinomas (*p* = 0.029) with no indication of heterogeneity across the studies. Reanalysis of the non-neoplastic toxicity data show there is a significant decrease in basophilic-cell foci in study K (*p* = 0.023), no foci at all in study L and no trend in study M. Clear-cell foci are not impacted by glyphosate in male Wistar rats.

Pituitary adenomas are increased in both males (*p* = 0.045) and females (*p* = 0.014) in study M but not in the remaining studies. Carcinomas show no increase in any study but the combined adenomas and carcinomas are marginally significant in males (*p* = 0.059) and significant in females (*p* = 0.017) in study M but not in the others. None of the pooled analyses for a common trend are statistically significant although the pooled trend in males is marginally significant for both adenomas (*p* = 0.057) and combined adenomas and carcinomas (*p* = 0.073). There are no dose-dependent increases in any non-neoplastic lesion in male or female Wistar rats in any of the three studies.

Skin keratoacanthomas are significantly increased in males in study M (*p* = 0.030) and in the pooled analysis for a common trend (*p* = 0.032) with no heterogeneity. There are no keratoacanthomas in study K and a slight increase with dose in study L. No non-neoplastic pathologies are significantly linked to dose in the skin.

Adrenal pheochromocytomas are increased in study K (*p* = 0.048) but not in the other studies or in the pooled analysis. There are no significant trends in non-neoplastic findings in any of the three studies.

Mammary gland adenomas (*p* = 0.062), adenocarcinomas (*p* = 0.042) and their combination (*p* = 0.007) are all increased in study M, but not in the remaining studies. There is a marginal increase in adenocarcinomas in the pooled analysis for a common trend (*p* = 0.071) but not for the combined tumors (*p* = 0.110). The data suggests that all three endpoints demonstrated heterogeneity. Studies L and M also have fibroadenomas as well as adenomas and adenocarcinomas. Combining fibroadenomas, adenomas and adenocarcinomas results in no significant findings in any study or in the pooled analysis for this combination. Hyperplasia in mammary tissue is examined in all three studies with no significant findings in any study.

### Related findings from the peer-reviewed literature

There are numerous studies in the literature that relate to the cancer findings shown in Tables 3, 4 and 5. Some of the studies are done using pure glyphosate, but many use a GBH and present the results in glyphosate-equivalent doses. GBHs contain adjuvants, some of which are also likely to be highly toxic. In what follows, these related studies are discussed and care is taken to note whether the exposure is to glyphosate or a GBH. Caution should be used in interpreting the results using the GBHs since, in most cases, it is not clear if the resulting toxicity is due to the glyphosate in the GBH or the adjuvant(s).

Increases in kidney adenomas and carcinomas (combined) are seen in male CD-1 mice and increases in adenomas are seen in Swiss albino mice and SD rats in the reanalysis in this review. A number of short-term toxicity studies have demonstrated damage to the kidneys in laboratory animals from exposure to glyphosate or GBHs. Turkman et al. [46] saw significant (*p* < 0.05) increases in malondialdehyde (MDA) levels and decreases in glutathione (GSH) levels in male Wistar albino rats exposed to the GBH Knockdown 48SL. They also saw degeneration in the tubulur epithelial cells and expansion and vacuolar degeneration in glomerulus Bowman’s capsule (*p* < 0.05 for both). Dedeke et al. [47] also saw significant changes in MDA, GSH and several other kidney biomarkers from exposure to the GBH Roundup in male albino rats. They also studied glyphosate alone in equal doses to the GBH and saw smaller, but still significant increases in MDA and GSH, but not in the other biomarkers. In addition, they found that the amount of glyphosate in kidney tissue was substantially higher from exposure to the GBH than from exposure to glyphosate alone. Tang et al. [48] saw proximal and distal tubular necrosis (*p* < 0.01), glomerular toxicity (*p* < 0.01) and a reduction in weight (*p* < 0.05) in the kidneys of male SD rats exposed to glyphosate. They used a histopathological score and saw significant changes (*p* < 0.01) even down to a dose of 5 mg/kg body weight. Hamdaoui et al. [49] saw numerous histological changes and changes in urine and plasma associated with renal disfunction in female Wistar rats exposed to the GBH Kalach 360 SL. Kidney damage included fragmented glomeruli, necrotic epithelial cells, and tubular dilatation, inflammation, proximal tubular necrosis and distal tubular necrosis. Tizhe et al. [50] also saw glomerular degeneration, mononuclear cell infiltration and tubular necrosis in male and female Wistar rats exposed to the GBH Bushfire. Cavusoglu et al. [51] saw similar changes in blood chemistry and kidney pathology in male albino mice exposed to the GBH Roundup Ultra-Max. Wang et al. [52] saw kidney damage to tubular cells in Vk*MYC mice exposed to glyphosate in water.

In humans, GBHs are suspected to be involved in chronic kidney disease of unknown etiology (CKDu) in Sri Lanka, Mexico, Nicaragua, El Salvador and India [53–55]. Finally, the English abstract of a Chinese article by Zhang et al. [56] describe significant increases (*p* < 0.05) in abnormal hepatorenal function in workers occupationally exposed to glyphosate from 5 glyphosate-producing factories.

Dose-related increases in malignant lymphomas are seen in male and female CD-1 mice and marginal increases are seen in male and female Swiss albino mice in the reanalysis presented here. Wang et al. [52] exposed male and female Vk*MYC mice from the C57Bl/6 genetic background to glyphosate (purity not provided) at an exposure of 1 g/L in drinking water for 72 weeks (approximately 18 months) with an appropriate control. In addition, using the same mice, 7-day exposures were given at doses of 0, 1, 5, 10 and 30 g/L of glyphosate (*n* = 5 per group). Glyphosate induced splenomegaly in both wild type (WT) and Vk*MYC mice. Both WT and Vk*MYC mice demonstrated a significant increase (*p* < 0.05) in IgG levels when compared to controls. Vk*MYC treated mice had a clear M-spike (an indicator of multiple myeloma - MM), WT mice had a weaker M-spike and no M-spike was detected in untreated animals regardless of genetics. In addition, there were multiple hematological abnormalities in treated versus untreated mice that were consistent with MM. Activation-induced cytidine deaminase (AID, a marker of *monoclonal gammopathy of undetermined significance* induction, a precursor of MM) was upregulated in both bone marrow and spleen of both Vk*MYC and WT mice in the 72-week study. The same upregulation in the spleen and bone marrow were seen in the 7-day exposure animals in a dose-dependent fashion. A smaller dose-dependent increase was seen in lymph nodes. This upregulation of AID supports an AID-mediated mutational mechanism for the induction of MM and malignant lymphoma in these mice.

In humans, GBHs have been shown to increase the risk ratios for non-Hodgkins lymphomas (NHL) in several meta-analyses [2, 57–59]. For over 30 years, mouse models have been studied and evaluated as surrogates for NHL [60–64]. Classification systems for humans and mice indicate a strong similarity between malignant lymphomas in mice and NHL in humans.

Skin keratoacanthomas are increased by glyphosate in male SD rats and male Wistar rats. Skin basal-cell tumors are also increased in male SD rats in the reanalysis in this review. George et al. [35] exposed Swiss Albino mice to a glyphosate formulation (Roundup Original, 36 g/L glyphosate) in a typical skin-painting initiation-promotion study using 12-o-tetradecanoylphorbol-13-acetate (TPA) as a promoter and 7,12-dimethyl-benz[a]anthracene (DMBA) as an initiator. The group exposed to DMBA followed by glyphosate demonstrated a significant increase (*p* < 0.05) in the number of animals with tumors (40% of the treated animals versus no tumors in the controls) indicating the GBH has a promotional effect on carcinogenesis in the two-stage model in skin. Several in-vitro studies using human skin cells [65–67] have shown an increase in oxidative stress following exposure to glyphosate.

This review shows hepatocellular adenomas are increased by exposure to glyphosate in male SD rats and Wistar rats. Glyphosate has been shown to affect energy metabolism of mitochondria [68–71] and AST, ALT, and LDH [72] but not peroxisome proliferation or hypolipidemia [73] in the livers of Wistar rats. Transcriptome analyses of liver tissue in Sprague-Dwaley rats chronically exposed to the GBH Roundup Grand Travaux Plus suggest liver tissue damage is occurring [74]. Glyphosate and GBHs also seem to induce oxidative stress in the livers of several rat strains [48, 75, 76].

Adrenal cortical carcinomas are increased in female Sprague-Dawley rats in the reanalysis in this review. There is also a suggestion of an increase in adrenal pheochromocytomas in male Wistar rats and of pituitary adenomas in male and female Wistar rats. Owagboriaye et al. [77] saw a significant increase in adrenal hormones aldostererone and corticosterone in a dose-dependent fashion following exposure to a GBH (Roundup Original) in male albino rats but not following exposure to equivalent doses of glyphosate (purity not given). Significant changes in adrenocorticotropic hormone were also seen for the GBH but not glyphosate. In contrast, Pandey and Rudraiah [78] saw a significant reduction in adreno-corticotropic hormone levels at similar doses in Wistar rats. Romano et al. (2010) saw a reduction in adrenal weights from exposure to the GBH Roundup Transorb in newly-weaned male Wistar rats but saw no differences in corticosterone levels except a rather large, non-statistical increase at the lowest exposure group. Changes in these and other hormones in these three papers suggest GBHs could have an impact on the hypothalamic-pituitary-adrenal axis that, after lifetime exposure, could induce cancers in the adrenal cortex and/or pituitary.

This reanalysis shows an inconsistent effect of glyphosate on the rates of mammary gland adenomas, carcinomas and combined adenomas and carcinomas in female Wistar rats but not in SD rats. Seralini et al. (2014) [36] saw an increase in mammary tumors in female SD rats exposed to the GBH GT Plus with associated hypertrophies and hyperplasia. Glyphosate and GBHs have also been shown to disrupt estrogen receptor alpha in rats [79] and to alter cellular replication and genotoxicity in estrogen-sensitive cell lines [80–86].

The longest study in male Sprague-Dawley rats showed an increase in testicular interstitial cell tumors after reanalysis. Several studies have seen changes in aromatase, testosterone and/or estrogen levels in male rats exposed to glyphosate or GBHs [84, 87–93].

The reanalysis in this review show an inconsistent increase in thyroid C-cell adenomas and/or carcinomas in male and female SD rats and thyroid follicular cell adenomas in male SD rats. De Souza et al. [94] exposed male Wistar rats to the GBH Roundup Transorb from gestational day18 to postnatal day 5 and examined the animals for thyroid hormone effects at postnatal day 90. They saw dose-dependent decreases in thyroid stimulating hormone but no changes in circulating triiodothyronine or thyroxine. Genomic analysis suggested that genes involved in thyroid hormone metabolism and transport were probably involved in these alterations. In humans, Samsel et al. [95] hypothesized that glyphosate intake could interfere with selenium uptake, impacting thyroid hormone synthesis and increasing thyroid cancer risks. Using data from the Agricultural Health Study, Shrestha et al. [96] saw an association between ever/never use by farmworkers of GBHs and hypothyroidism (OR = 1.28, 95% CI 1.07–1.52) and for the two lowest categories of intensity of use, but not the highest category.

### False positive errors

The evaluation of any one animal cancer study involves a large number of statistical tests that could lead to false positives. To evaluate this issue, the probability that all of the results in any sex/species/strain could be due to false positive results is calculated. Overall, a total of 496 evaluations are done for these 13 studies including the few evaluations done against historical controls. There are 41 evaluations at 37 tumor/site combinations with a trend test *p* ≤ 0.05; the probability that all of these are due to false positives is 0.001. Similarly, looking at the evaluations resulting in *p* ≤ 0.01, the probability that all of the findings are due to false positives is < 0.001. The strongest evidence is for male CD-1 mice, the probability of seeing 11 positive findings at *p* ≤ 0.05 and 8 at *p* ≤ 0.01 are both below 0.001. (see Additional file 2: Table S14).

### Comparison to regulator reviews

In their final report on the carcinogenicity of glyphosate, the EPA concluded that “*Based on the weight-of-evidence evaluations, the agency has concluded that none of the tumors evaluated in individual rat and mouse carcinogenicity studies are treatment-related due to lack of pairwise statistical significance, lack of a monotonic dose response, absence of preneoplastic or related non-neoplastic lesions, no evidence of tumor progression, and/or historical control information (when available). Tumors seen in individual rat and mouse studies were also not reproduced in other studies, including those conducted in the same animal species and strain at similar or higher doses*.” EFSA concluded “*No evidence of carcinogenicity was confirmed by the large majority of the experts (with the exception of one minority view) in either rats or mice due to a lack of statistical significance in pair-wise comparison tests, lack of consistency in multiple animal studies and slightly increased incidences only at dose levels at or above the limit dose/MTD, lack of pre-neoplastic lesions and/or being within historical control range. The statistical significance found in trend analysis (but not in pair-wise comparison) per se was balanced against the former considerations.”* Other regulatory agencies used similar wording to describe their findings. Each of the issues cited in these summaries are discussed below.

Both EPA and EFSA describe a lack of significant pairwise comparisons as one reason for discarding positive findings due to positive trend analyses. This is in direct conflict with their guidelines [38, 39] which make it clear that a positive finding in either pairwise comparisons or trend tests should be sufficient to rule out chance. The net effect of requiring both tests to be positive is an increase the probability of a false negative finding.

EPA notes that a lack of monotonic dose-response was a factor in their evaluation and, even though not mentioned in EFSA’s final conclusions, was also used by EFSA to eliminate positive findings. This restriction suggests a serious lack of understanding of statistical variation in tumor responses and the way in which trend tests treat this variation, especially when the lowest doses are close to the control response and the increased tumor response is low. The net effect of requiring monotonic dose-response is a severe reduction in the ability to detect a positive trend and a large increase in the probability of a false negative finding.

Both agencies note that a lack of preneoplastic or related non-neoplastic lesions led to the exclusion of some tumors. For some of the tumors mentioned above, this is the case, but certainly not for all of them as noted in the analyses shown in Tables 3, 4 and 5. In addition, both agencies failed to evaluate support in the scientific literature for any of the tumors and relied entirely on the cancer bioassay results alone to draw any conclusions. In this evaluation, changes in preneoplastic and non-neoplastic conditions are analyzed for all tissues showing positive tumor findings and in all studies with the same sex/species/strain using an appropriate trend test and many tissue changes that could relate to these tumors are identified.

Both EPA and EFSA noted that historical controls are used in their evaluations. However, in both cases, the agencies only cite the range of the historical controls as a factor when determining if a given positive cancer finding is caused by glyphosate. As noted by the IARC [40] “*It is generally not appropriate to discount a tumour response that is significantly increased compared with concurrent controls by arguing that it falls within the range of historical controls*.” In general, the concurrent control group is the most appropriate for any statistical analysis of the data [38–40], however, historical controls can play an important role in evaluating changes in rare tumors and cases where it appears the control response is unreasonably low and the treated groups appear to be unchanged from each other and in the central area of the historical control data. In this evaluation, a formal statistical test [41] is used to evaluate the cancer data when it is appropriate to use historical controls rather than inappropriately using only the historical control range. In addition, in every case where EPA and EFSA noted a significant tumor response was in the range of the historical control data, the reanalysis in this paper using Tarone’s test demonstrates greater statistical significance in the trend and in no case invalidates a positive trend (not shown for all cases).

EPA cites no evidence of tumor progression as a reason to exclude some of the cancer findings. For some tumors, such as malignant lymphomas, tumor progression is not an issue. In cases where there is clearly tumor progression such as for mammary gland adenomas and adenocarcinomas in study M, the agency did not consider this progression to be compelling. In addition, in cases where there is a clear increase in carcinomas and a slight decrease in adenomas, as might occur if the chemical impacts a later stage in the carcinogenic process or is a promoter, the agency did not consider this possibility. Similar comments apply to EFSA’s evaluation.

EFSA notes that many studies had positive findings at or above the limit dose/MTD as a reason for excluding many study findings. There is clear guidance in the literature and regulatory guidelines on what constitutes exceedance of the MTD and how to exclude these data [39, 40, 97]. In no case did EFSA or EPA conclude that the highest dose used in any study they reviewed exceeded the MTD. The limit dose derives from the OECD guidelines for combined chronic toxicity/carcinogenicity studies [98] which states that “*For the chronic toxicity phase of the study, a full study using three dose levels may not be considered necessary, if it can be anticipated that a test at one dose level, equivalent to at least 1000 mg/kg body weight/day, is unlikely to produce adverse effects.*” It is difficult to understand how a finding of carcinogenicity at a dose above 1000 mg/kg/day can be excluded based upon this guidance if that dose does not exceed the MTD.

Both EFSA and EPA found that there was inconsistency between studies of the tumor response and used this reasoning to exclude several tumors. Part of this relates to findings appearing in only one sex or strain but not others; this happens quite often, for example see [99] for animal carcinogenicity findings for 111 known human carcinogens. The other part of this relates to the magnitude of the response in a specific sex/species/strain; neither agency used a formal statistical method to evaluate this consistency. It is naive to assume that the raw tumor counts from studies done in different laboratories at different times using different diets, different exposure lengths and different sub-strains of animals would yield perfect agreement in response. EPA’s FIFRA Science Advisory Panel, in their review of EPA’s draft risk assessment [100] recommended EPA do a pooled analysis to determine an overall effect as does the IARC [40]. The pooled analyses presented in this evaluation properly adjust for study differences and demonstrate consistency for many of the tumors showing significant evidence of carcinogenicity in one or more studies and suggestive increases in carcinogenicity in other studies using the same sex/species/strain.

Finally, both agencies missed many of the tumors identified in this evaluation due to a failure to analyze all of the data using a trend test like the C-A test. EPA states that in 4 of the 8 rat carcinogenicity studies no tumors were identified for evaluation. For one of these studies [30], the data are unavailable for review and the doses are far below the MTD. For the remaining three studies [19–21], there are 5 positive findings not identified by the EPA. In the remaining 4 studies [17, 18, 22, 23] where they saw some tumors increased, they failed to identify 6 tumors identified in this reanalysis. EPA states that in 2 of the 6 mouse carcinogenicity studies no tumors were identified for evaluation. As noted in the Materials and methods section, one of these studies [24] was determined to have falsified data by EPA [25] and should not have been included in their evaluation. For the second study [26], the data are unavailable and could not be evaluated in this review. In the remaining four studies discussed by EPA [11–14], they missed 5 tumors identified in this evaluation (two identified through historical controls). In addition, they excluded one study [16] due to the presence of a viral infection within the colony; EPA gives no documentation of this viral infection and there is no indication within the study report of a viral infection nor any indication that these animals were unhealthy. This study has one significant finding not discussed by EPA and three marginally significant findings similar to those seen in CD-1 mice. EPA also failed to evaluate one study [13] considered in this evaluation which had two positive tumor findings. Thus, EPA discussed only 7 of the 21 statistically significant tumor increases in rats and 5 of the 16 significant tumor increases in mice. Similar comments apply to the EFSA review and all of the other regulatory reviews. To be fair to the regulatory agencies, it should be noted that the original study reports from the laboratories that did these studies also failed to identify many of the significant trends discussed in this review because they relied predominantly on pairwise evaluations like Fisher’s exact test and failed to do any trend analyses. This would suggest that the regulatory agencies are relying upon the results of the analyses presented in the study reports rather than conducting their own thorough reanalysis of the data using trend tests.

The mechanisms through which glyphosate causes these tumors in laboratory animals are as controversial as the cancer findings themselves. The IARC Working Group [2] concluded there was strong evidence that glyphosate induces genotoxicity and oxidative stress. All of the regulatory reviews have concluded glyphosate is not genotoxic and most have concluded it does not cause oxidative stress. A complete review of this literature is beyond the scope of this manuscript, but as noted above, genotoxicity and oxidative stress are plausible mechanisms for many of these cancers. Also, as noted in the earlier discussion of related findings from the peer-reviewed literature, some of the cancers may be due to glyphosate altering hormonal balance in the adrenal, pituitary and thyroid glands.

### Strength-of evidence conclusions

In summary, exposure of rats and mice to glyphosate in 13 separate carcinogenicity studies demonstrates that glyphosate causes a variety of tumors that differ by sex, species, strain and length of exposure. To summarize the strength-of-evidence for each tumor, four categories are used. Clear evidence (CE) is indicated when the data demonstrate a causal linkage between glyphosate and the tumor based upon the reanalysis in this review and the available peer-reviewed literature. Some evidence (SE) is indicated when the data demonstrate a linkage between glyphosate and the tumor based upon the reanalysis in this review and the available peer-reviewed literature but chance, although unlikely, cannot be ruled out. Equivocal evidence (EE) also indicates the data demonstrate a linkage between glyphosate and the tumor based upon the reanalysis in this review and the available peer-reviewed literature, but chance is as likely an explanation for the association as is glyphosate. No evidence (NE) indicates any linkage between glyphosate and the tumor based upon the reanalysis in this review is almost certainly due to chance. The factors used to put tumors into these categories include the analyses of the individual studies, the consistency of the data across studies (the pooled analyses), the analyses using historical control data, the analyses of the non-neoplastic lesions, the mechanistic evidence and the associated scientific literature. These categorizations are presented in Table 6.

**Table 6:** Summary of level of evidence^a^ for tumors observed to have a significant trend in 13 rodent carcinogenicity studies in male and female, mice and rats^b^

Tumor	Males				Females			
	CD-1 Mouse	Swiss Mouse	SD Rat	Wistar Rat	CD-1 Mouse	Swiss albino mouse	SD Rat	Wistar Rat
Adrenal cortical carcinoma							CE	
Adrenal pheochromocytoma								EE
Alviolar-Bronchiolar tumor	NE				NE			
Harderian gland tumor					NE			
Hemangioma					CE	CE		
Hemangiosarcomas	CE							
Kidney tumor	CE	SE	CE					
Liver adenoma			CE	CE				
Mammary tumor								SE
Malignant lymphoma	CE	SE			CE	SE		
Pancreas Islet Cell tumor			EE					
Pituitary adenomas				SE				SE
Skin basal-cell tumor			CE					
Skin keratoacanthoma			CE	CE				
Thyroid C-cell tumor			EE				EE	
Thyroid follicular-cell tumor			EE					
Testis interstitial-cell Tumor			SE					

^a^*CE* Clear evidence, *SE* Some evidence, *EE* Equivocal evidence, *NE* No evidence: ^b^a blank space indicates there is no positive finding in any study for this tumor in this sex/species

There is clear evidence that glyphosate causes hemangiosarcomas, kidney tumors and malignant lymphomas in male CD-1 mice and hemangiomas and malignant lymphomas in female CD-1 mice. There is clear evidence that glyphosate causes hemangiomas in female Swiss albino mice. There is clear evidence that glyphosate causes kidney adenomas, liver adenomas, skin keratoacanthomas and skin basal cell tumors in male Sprague-Dawley rats and adrenal cortical carcinomas in female Sprague-Dawley rats. There is clear evidence that glyphosate causes hepatocellular adenomas and skin keratocanthomas in male Wistar rats.

There is some evidence that glyphosate causes malignant lymphomas in male and female and kidney tumors in male Swiss albino mice. There is some evidence that glyphosate causes testicular interstitial cell tumors in male Sprague-Dawley rats. There is some evidence that glyphosate causes pituitary adenomas in male and female Wistar rats and mammary gland adenomas and carcinomas in female Wistar rats.

There is equivocal evidence that glyphosate causes thyroid c-cell adenomas and carcinomas in male and female Sprague-Dawley rats, and thyroid follicular cell adenomas and carcinomas and pancreas islet-cell adenomas in male Sprague-Dawley rats. There is equivocal evidence glyphosate causes adrenal pheochromocytomas in male Wistar rats.

There is no evidence that glyphosate causes lung tumors in male and female CD-1 mice or Harderian gland tumors in female CD-1 mice.

The analyses conducted for this review clearly support the IARC’s conclusion that there is sufficient evidence to say that glyphosate causes cancer in experimental animals. In contrast, the regulatory authorities reviewing these data appear to have relied on analyses conducted by the registrant and not their own analyses of the data. As such, they uniformly concluded that the subset of tumor increases they identified as showing an association with glyphosate were due to chance. Had regulatory authorities conducted a full reanalysis of all of the available evidence from the 13 animal carcinogenicity studies as was done here, it is difficult to see how they could reach any conclusion other than glyphosate can cause cancers in experimental animals.

## Supplementary information

**Additional file 1.** Details on individual animal chronic exposure toxicity and carcinogenicity studies.

**Additional file 2: Table S1.** Tumors of interest in male and female CD-1 mice from the 24-month feeding study of Knezevich and Hogan (1983) [11] – Study A. **Table S2.** Tumors of interest in male and female CD-1 mice from the 24-month feeding study of Atkinson et al. (1993) [12] – Study B. **Table S3.** Tumors of interest in male and female CD-1 mice from the 18-month feeding study of Sugimoto (1997) [13] – Study C. **Table S4.** Tumors of interest in male and female CD-1 mice from the 18-month feeding study of Wood et al. (2009) [14] – Study D. **Table S5.** Tumors of interest in male and female CD-1 mice from the 18-month feeding study of Takahashi (1999) [15]; data extracted from JMPR [7] – Study E. **Table S6.** Tumors of interest in male and female Swiss Albino mice from the 18-month feeding study of Kumar (2001) [16] – Study F. **Table S7.** Tumors of interest in male and female Sprague-Dawley rats the 26-month feeding study of Lankas (1981) [17] – Study G. **Table S8.** Tumors of interest in male and female Sprague-Dawley rats from the 24-month feeding study of Stout and Ruecker (1990) [18] – Study H. **Table S9**. Tumors of interest in male and female Sprague-Dawley rats from the 24-month feeding study of Atkinson et al. (1993) [19] – Study I. **Table S10.** Tumors of interest in male and female Sprague-Dawley rats from the 24-month feeding study of Enemoto (1997) [20] – Study J. **Table S11.** Tumors of interest in male and female Wistar rats from the 24-month feeding study of Suresh (1996) [21] – Study K. **Table S12.** Tumors of interest in male and female Wistar rats from the 24-month feeding study of Brammer (2001) [22] – Study L. **Table S13.** Tumors of interest in male and female Wistar rats from the 24-month feeding study of Wood et al. (2009) [23] – Study M. **Table S14.** Observed (Obs.) versus expected (Exp.) tumor sites with significant trends in the 13 acceptable rodent carcinogenicity studies using glyphosate.

## Data Availability

The original reports for 12 of the animal carcinogenicity studies that support the findings of this study are available from EFSA, but restrictions apply to the availability of these data. All tumor data cited in this study are included in this published article [and its supplementary information files]. Additional data (historical control data, non-significant cancer sites, non-neoplastic endpoints, etc.) are available from the author upon reasonable request.

## Abbreviations

AID: Activation-induced cytidine deaminase
ALT: Alanine aminotransferase
AST: Aspartate aminotransferase
DMBA: 7,12-dimethyl-benz[a]anthracene
EChA: European Chemicals Agency
EFSA: European Food Safety Authority
EPA: US Environmental Protection Agency
GBH: Glyphosate-based herbicide
GSH: Glutathione
IARC: International Agency for Research on Cancer
JMPR: Joint Meeting of the FAO Panel of Experts on Pesticide Residues in Food and the Environment and the WHO Core Assessment Group on Pesticide Residues
LDH: Lactic acid dehydrogenase
MDA: Malondialdehyde
mg/kg/d: Milligrams per kilogram body weight per day
MM: Multiple myeloma
MTD: Maximum tolerated dose
OECD: Organization for Economic Cooperation and Development
SD rat: Sprague-Dawley rat
TPA: 12-o-tetradecanoylphorbol-13-acetate
WT: Wild type
