# Supplementary material for: A comprehensive analysis of the animal carcinogenicity data for glyphosate from chronic exposure rodent carcinogenicity studies
Source: Environ Health. 2020 Feb 12;19:18. doi: 10.1186/s12940-020-00574-1 (PMC7014589; doi:10.1186/s12940-020-00574-1)
Supplement: Supplementary file 1 — Additional file 1. Details on individual animal chronic exposure toxicity and carcinogenicity studies. [file 12940_2020_574_MOESM1_ESM.docx]

**Additional file 1**

Details on individual animal chronic exposure toxicity and carcinogenicity studies

*Knezevich and Hogan (1983) [11] – Study A*

**Title:** A Chronic Feeding Study of Glyphosate (Roundup Technical) in Mice

**Laboratory:** BIO/Dynamics Inc., East Millstone, NJ

**In-Life Dates:** 31 March, 1980 to 8 March, 1982

**Guidelines Used:** Unknown

**Test material:** Glyphosate Technical

**Purity:** 99.7%

**Route of Exposure:** Diet

**Species:** Mouse

**Strain:** Charles River CD-1, COBS (ICR derived)

**Age at start:** 40 days

**Sex:** M and F

**Diet:** Purina Rodent Laboratory Chow #5001

**Housing:** Individually housed in stainless steel wire mesh cages

**Dosing:** 0, 1,000, 5,000 and 30,000 ppm

**Animals per group (main study):** 50

**Duration of exposure (main study):** 24 months

**Interim Sacrifice:** None

**Number of animals per group (interim sacrifice):** None

**Tissues examined:** adrenals, aorta (abdominal), bone & bone marrow (costochondral junction), brain (3 sections), caecum, colon, duodenum, epididymides, eyes (with optic nerve), gross lesions including palpable masses, head, heart, Harderian gland, ileum, jejunum, kidneys, liver, lungs, lymph nodes (mediastinal and mesenteric), mammary gland, muscle (skeletal), oesophagus, ovaries, pancreas, pituitary, prostrate, rectum, mandibular salivary glands, sciatic nerve, seminal vesicles, skin, skeletal muscle, spinal cord (cervical), spleen, stomach, testes, thymus, thyroid/parathyroid, tongue, trachea, urinary bladder, and uterus

**Pathology protocol:** All animals, all tissues except spinal cord and head which were examined for 10 animals in each group.

**Tumors evaluated in this reanalysis:**

**Males:** Adrenal Gland: Cortical adenoma; Kidney: Renal tubule adenoma (original and revised), carcinoma (revised), combined (revised); Liver: Hepatocellular adenoma or carcinoma, Hepatocellular adenocarcinoma; Lung: Alveolar/Bronchiolar Adenoma, Adenocarcinoma, combined; Lymph Node, Mediastinal: Lymphoblastic Lymphosarcoma with leukemic manifestations; Lymph Node, Mesenteric: Lymphoblastic Lymphosarcoma with leukemic manifestations; Spleen: Lymphoblastic Lymphosarcoma with leukemic manifestations; Systemic Tumors: Malignant lymphoma, Hemangiosarcoma; Testes: Interstitial cell tumor,

**Total number of trend tests: 19 (3 historical control)**

**Females:** Harderian Gland: Adenoma, Carcinoma, combined; Liver: Hepatocellular adenocarcinoma; Lung: Alveolar/bronchiolar adenoma, adenocarcinoma, combined; Lymph Node, Mediastinal: Lymphoblastic Lymphosarcoma with leukemic manifestations; Lymph Node, Mesenteric: Composite Lymphosarcoma, Lymphoblastic Lymphosarcoma with leukemic manifestations; Mammary Gland: Ductal adenocarcinoma; Spleen: Hemangioendothelioma, Granulocytic Leukemia, Composite Lymphosarcoma (positive, not shown in tables); Systemic Tumors: Malignant Lymphoma, Hemangiomas; Uterus: Leiomyoma, Leiomyosarcoma, Endometrial adenocarcinoma

**Total number of trend tests: 19**

*Atkinson et al. (1993) [12] – Study B*

**Title:** Glyphosate 104 Week Dietary Carcinogenicity Study in Mice

**Laboratory:** Inveresk Research International Limited, Elphinstone Research Center, Tranent, Scotland

**In-Life Dates:** 20 December, 1989 to 23 December, 1991

**Guidelines Used:** OECD, USEPA, Japanese MAFF

**Test material:** Glyphosate Technical

**Purity:** 97.5%

**Route of Exposure:** Diet

**Species:** Mouse

**Strain:** Charles River CD-1

**Age at start:** approximately 7 weeks

**Sex:** Male and Female

**Diet:** Finely ground #1 Diet SQC Expanded from Special Diets Services Limited, Essex

**Housing:** One animal per cage, suspended polypropylene cages

**Dosing:** 0, 100, 300 and 1,000 mg/kg/day

**Animals per group (main study):** 50

**Duration of exposure (main study):** 104 weeks

**Interim Sacrifice:** None

**Number of animals per group (interim sacrifice):** NA

**Tissues examined:** adrenals, aorta, bladder, bone, brain, caecum, colon, duodenum, eyes (with optic nerve), gross lesions including palpable masses, heart, ileum, jejunum, kidneys, liver, lungs, lymph nodes (submandibular and mesenteric), mammary gland, muscle (skeletal), oesophagus, ovaries, pancreas, pituitary, prostrate, rectum, salivary glands, sciatic nerve, seminal vesicles, skin, skeletal muscle, spinal cord (cervical, mid-thoracis, lumbar), spleen, stomach, testes, thymus, thyroid/parathyroid, tongue, trachea, urinary bladder, uterus and vagina

**Pathology protocol:** Histopathological examination was carried out on all tissues collected from control and high dose animals, all animals that died or were killed in extremis during the study, all gross and palpable lesions, livers, lungs, kidneys and bone marrow of all animals.

**Tumors evaluated in this reanalysis:**

**Males:** Adrenal: Cortical adenoma, carcinoma, combined; Kidney: Tubular adenoma, carcinoma, combined; Liver: Hepatocellular Adenoma, Carcinoma, combined; Lung: Alveolar/Bronchiolar adenoma, carcinoma, combined; Lymphoreticular tissue: Histiocytic Sarcoma, Malignant lymphoma; Testes: Interstitial cell adenoma; Vascular System: Hemangiosarcoma,

**Total number of trend tests: 16**

**Females:** Kidney: Tubular adenoma or carcinoma; Liver: Hepatocellular Adenoma, Carcinoma, combined; Lung: Alveolar/Bronchiolar adenoma, carcinoma, combined; Lymphoreticular tissue: Histiocytic Sarcoma, Malignant lymphoma; Pituitary Gland: Adenoma; Skin/Subcutis: Sarcoma, unknown origin; Vascular System: Hemangiosarcoma, Hemangioma

**Total number of trend tests: 13**

*Sugimoto (1997) [13] – Study C*

**Title:** 18-Month Oral Oncogenicity Study in Mice

**Laboratory:** The Institute of Environmental Toxicology, Tokyo, Japan

**In-Life Dates:** 21 February, 1995 to 6 September, 1996

**Guidelines Used:** Japan MAFF Guidelines 59 NohSan No.4200, 1985, U.S. EPA FIFRA Guidelines Subdivision F, 1984, OECD 451 (1981).

**Test material:** Glyphosate Technical

**Purity:** 94.61% to 97.56%

**Route of Exposure:** Diet

**Species:** Mouse

**Strain:** SPF ICR (Crj:CD-1)

**Age at start:** 5 weeks

**Sex:** Males and Females

**Diet:** Certified diet MF Mash, Oriental Yeast Co., Ltd.

**Housing:**

**Dosing:** 0, 1,600, 8,000 and 40,000 ppm

**Animals per group (main study):** 50

**Duration of exposure (main study):** 18 months

**Interim Sacrifice:**

**Number of animals per group (interim sacrifice):**

**Tissues examined:** brain, spinal cord, sciatic nerve, pituitary, thymus, thyroids

with parathyroids, adrenals, spleen, bone with marrow, tibio-femoral joint, lymph nodes,

heart, aorta, salivary glands, esophagus, stomach, liver with gallblader , pancreas, duodenum,

jejunum, ileum, cecum, colon, rectum, trachea, lung, kidneys, urinary bladder, testes, prostate, seminal vesicles, epididymides, coagulating glands, ovaries, uterus, vagina, harderian glands, eyes, skeletal muscle, skin, mammary gland, all gross lesions

**Pathology protocol:** A detailed histopathological examination was performed on all sampled tissues in all dose groups.

**Tumors evaluated in this reanalysis:**

**Males:** Harderian Gland: Adenoma, Hematopoetic: Malignant lymphoma; Kidney: Adenoma; Liver: Hepatocellular adenoma, carcinoma, combined; Lung: Adenoma, Adenocarcinoma, combined; Systemic:Hemangiosarcoma,

**Total number of trend tests: 13 (3 historical controls)**

**Females:** Adrenal: A-cell tumor, Pheochromocytoma; Harderian Gland: Adenoma; Hematopoetic: Malignant lymphoma; Liver: Hepatocellular Adenoma; Lung: adenoma, adenocarcinoma, combined; Mammary Gland: adenoma, adenocarcinoma, combined; Systemic: Hemangioma; Uterus: Hemangioma, Leiomyoma, Leiomyosarcoma

**Total number of trend tests: 15**

*Wood et al.* *(2009) [14] – Study D*

**Title:** Glyphosate technical: Dietary Carcinogenicity Study in the Mouse

**Laboratory:** Harlan Laboratories Limited, Shardlow, UK

**In-Life Dates:** 10 October, 2005 to 19 November, 2007

**Guidelines Used:** OECD 451 (1981), JMAFF guideline 2-1-15 (2005), US-EPA OPPTS 870.4200 (1996)

**Test material:** Glyphosate Technical

**Purity:** 95.7%

**Route of Exposure:** Diet

**Species:** Mouse

**Strain:** Crl:CD-1 (ICR) BR

**Age at start:** 5-6 weeks

**Sex:** Male and Female

**Diet:** SQC Ground diet No. 1, Special Diet Services Limited, UK

**Housing:** Three per cage by sex, polypropylene solid-floor cages

**Dosing:** 0, 500, 1,500, 5,000 ppm

**Animals per group (main study):** 51

**Duration of exposure (main study):** 79 weeks

**Interim Sacrifice:** 39 Weeks

**Number of animals per group (interim sacrifice):** 10

**Tissues examined:** adrenals, aorta (thoracic), bone & bone marrow (sternum and femur (incl. stifle joint)), brain (incl. cerebrum, cerebellum and pons), caecum, colon, duodenum, epididymides, eyes (with optic nerve), gross lesions incl. palpable masses, head (incl. pharynx, nasopharynx and paranasal sinuses), heart, Harderian and lacrimal glands, ileum, jejunum, kidneys, larynx, liver and gall bladder, lungs (with bronchi), mammary gland, lymph nodes (cervical and mesenteric), muscle (skeletal), oesophagus, ovaries, pancreas, pituitary, preputial gland, prostrate, rectum, salivary glands, sciatic nerve, seminal vesicles, skin (hind limb), spinal cord (cervical, mid-thoracic and lumbar), spleen, stomach, testes, thymus, thyroid/parathyroid, tongue, trachea, urinary bladder, uterus and vagina

**Pathology protocol:** Histopathological examination was carried out on all tissues collected from control and high dose animals, all animals that died or were killed in extremis during the study, all gross and palpable lesions, livers, lungs, kidneys and bone marrow of all animals.

**Tumors evaluated in this reanalysis:**

**Males:** Kidney: Adenoma, Carcinoma, combined, Liver: Hepatocellular adenoma, carcinoma, combined; Lung: Adenoma, Adenocarcinoma, combined; Lymphoid/Hematopoetic: Malignant lymphoma; Skin: Fibrosarcoma; Systemic: Hemangiosarcoma

**Total number of trend tests: 12**

**Females:** Harderian Gland: Adenoma, Adenocarcinoma, combined; Lung: Adenoma, Adenocarcinoma, combined; Lymph Node, Mesenteric: Histiocytic sarcoma; Lymphoid/Hematopoetic: Histiocytic sarcoma, malignant lymphoma; Ovary: Luteoma; Pituitary Gland: Adenoma; Systemic: Hemangioma; Uterus: Endometrial stromal polyp, Histiocytic sarcoma

**Total number of trend tests: 15**

*Takahashi (1999) [15] – Study E*

**Title:** Oral feeding carcinogenicity study in mice with AK-01

**Laboratory:** Nippon Experimental Medical Research Institute Co. Ltd., Agatsuma, Gunma, Japan

**In-Life Dates:** Unknown

**Guidelines Used:** Unknown

**Test material:** Glyphosate Technical

**Purity:** 97.5%

**Route of Exposure:** Diet

**Species:** Mouse

**Strain:** Crj:CD-1

**Age at start:** Unknown

**Sex:** Males and Females

**Diet:** Unknown

**Housing:** Unknown

**Dosing:** 0, 500, 5,000, 50,000 ppm

**Animals per group (main study):** 50

**Duration of exposure (main study):** 78 weeks

**Interim Sacrifice:** Unknown

**Number of animals per group (interim sacrifice):** NA

**Tissues examined:** Unknown

**Pathology protocol:** Histopathological examination was carried out on all tissues collected from control and high dose animals, all animals that died or were killed in extremis during the study, all gross and palpable lesions.

**Tumors evaluated in this reanalysis:**

**Males:** Kidney: Adenoma, Carcinoma, combined

**Total number of trend tests: 3**

**Females:** malignant lymphoma

**Total number of trend tests: 1**

*Kumar (2001) [16] – Study F*

**Title:** Carcinogenicity Study with Glyphosate Technical in Swiss Albino Mice

**Laboratory:** Rallis Research Centre, Peenya, Bangalore, India

**In-Life Dates:** 18 December, 1997 to 29 June, 1999

**Guidelines Used:** OECD 451 (1981)

**Test material:** Glyphosate Technical

**Purity:** >95%

**Route of Exposure:** Diet

**Species:** Mouse

**Strain:** Swiss albino, HsdOla: MF1

**Age at start:** 6 weeks

**Sex:** Male and Female

**Diet:** Ssniff rat/mouse powder food maintenance meal – low in germs, M/s Ssniff Spezialdiäten, D-59494 Soest, Germany

**Housing:** 5 per cage, by sex, polypropylene cages

**Dosing:** 0, 100, 1,000 and 10,000 ppm

**Animals per group (main study):** 50

**Duration of exposure (main study):** 18 months

**Interim Sacrifice:** None

**Number of animals per group (interim sacrifice):** NA

**Tissues examined:** adrenals, bone & bone marrow (sternum and femur (incl. joint)), brain (incl. cerebrum, cerebellum pons), caecum, colon, duodenum, epididymides, eyes (with optic nerve), heart, jejunum, kidneys, larynx, liver and gall bladder, lungs, lymph nodes (mandibular, mesenteric, and superficial inguinal), muscle (femoral), oesophagus, ovaries, pancreas, pituitary, prostrate, rectum, salivary glands, sciatic nerve, seminal vesicles and coagulating glands, skin, spinal cord (cervical, mid-thoracic and lumbar), spleen, stomach, testes, thymus, thyroid/parathyroid, trachea, urinary bladder, uterus and all lesions and tumours/masses

**Pathology protocol:** Histopathological examination was carried out on all tissues collected from control and high dose animals, all animals that died or were killed in extremis during the study, all gross and palpable lesions

**Tumors evaluated in this reanalysis:**

**Males:** Lympho/Hemolymphoreticular system: Histiocytic sarcoma, malignant lymphoma, Myeloid leukemia, Kidney: Renal cell adenoma, carcinoma, combined; Liver: Hepatocellular adenoma, carcinoma, combined; Lung: Bronchio-alveolar adenoma, carcinoma, combined; Systemic: Hemangiosarcoma, Hemangioma

**Total number of trend tests: 14**

**Females:** Bone: Osteoma; Hemolymphoreticular system: Histiocytic sarcoma, malignant lymphoma, Myeloid leukemia; Liver: Hepatocellular adenoma, carcinoma, combined; Lung: Bronchio-alveolar adenoma, carcinoma, combined; Lymph Node, Mesenteric: Hemangioma; Ovaries: Endometrial stromal sarcoma; Systemic: Hemangioma; Uterus: Endometrial stromal sarcoma

**Total number of trend tests: 14**

*Lankas (1981) [17] – Study G*

**Title:** A Lifetime Feeding Study of Glyphosate (Roundup Technical) in Rats

**Laboratory:** BIO/Dynamics Inc., East Millstone, NJ

**In-Life Dates:** 12 July, 1978 to 4 September, 1980

**Guidelines Used:** Not stated. In accordance with OECD 453 (1981) according to BFR

**Test material:** Glyphosate Technical

**Purity:** 98.7%

**Route of Exposure:** Diet

**Species:** Rat

**Strain:** Sprague Dawley from Charles River Breeding Labs, Wilmington, Mass.

**Age at start:** 41 days

**Sex:** Males and Females

**Diet:** Purina Lab Chow

**Housing:** Individual animals in stainless steel cages

**Dosing:** 0, 30, 100 and 300 ppm

**Animals per group (main study):** 50

**Duration of exposure (main study):** 775-776 days (males), 784-785 days (females)

**Interim Sacrifice:** None

**Number of animals per group (interim sacrifice):** NA

**Tissues examined:** adrenals, aorta, blood smears, bone & bone marrow (costochondral junction), brain (cerebrum, cerebellum, brainstem), caecum, cervix, colon, duodenum, epididymis, eyes (retina, optic nerve), gross lesions including palpable masses, Harderian gland, heart, intestines (Including the caecum, colon, duodenum, ileum and jejunum), kidneys, lachrymal gland, larynx, liver, lung, lymph nodes (cervical and mesenteric), mammary gland, muscle, oesophagus, ovary, pancreas, pharynx, pituitary, prostrate, rectum, salivary glands (submandibular, parotid), seminal vesicles, skin, spinal cord (cervical, thoracic, lumbar), spleen, sternum, stomach, testes, thymus, thyroid/parathyroid, trachea, urinary bladder, uterus and vagina

**Pathology protocol:** A detailed histopathological examination was performed on all sampled tissues in all dose groups.

**Tumors evaluated in this reanalysis:**

**Males:** Adrenals: Pheochromocytoma, Cortical adenoma; Bone Marrow (Rib): Reticulum cell sarcoma; Brain: Glioma; Hind Foot: Osteoma; Kidney: Tubular adenoma, Lipoma; Liver: Reticulum cell sarcoma, Neoplastic nodule, Hepatocellular carcinoma, combined; Lung: Reticulum cell sarcoma; Lymph Node, Mesenteric: Angioma, malignant lymphoma; Mammary Gland: Galactocele; Pancreas: Islet cell adenoma, carcinoma, combined, Reticulum cell sarcoma; Pituitary Gland: Adenoma, Carcinoma, combined; Skin: Keratoacanthoma, Basosquamous cell tumor; Spleen: Reticulum cell sarcoma; Subcutis: Fibrosarcoma, Fibroma, Reticulum cell sarcoma, Lipoma; Testes: Interstitial cell tumor; Thyroid Gland: C-cell adenoma, carcinoma, combined, Follicular cell adenoma, carcinoma, combined;

**Total number of trend tests: 36**

**Females:** Adrenals: Reticulum cell sarcoma, Pheochromocytoma, Cortical adenoma, carcinoma, combined; Bone Marrow (Rib): Reticulum cell sarcoma; Kidney: Reticulum cell sarcoma; Liver: Reticulum cell sarcoma, malignant lymphoma, Hepatocellular carcinoma, Neoplastic nodule, combined; Lung: Reticulum cell sarcoma; Lymph Node, Mediastinal: Reticulum cell sarcoma, malignant lymphoma; Mammary Gland: Adenoma, Fibroadenoma, Adenocarcinoma, combined; Ovary: Granulosa cell tumor; Pancreas: Islet cell adenoma, carcinoma, combined; Pituitary Gland: Adenoma, Carcinoma, combined; Spleen: malignant lymphoma, Reticulum cell sarcoma; Thyroid Gland: C-cell adenoma, carcinoma, combined, Follicular cell, adenoma; Uterus: Adenoma, Polyp

**Total number of trend tests: 33**

*Stout and Ruecker (1990) [18] – Study H*

**Title:** Chronic Study of Glyphosate Administered in Feed to Albino Rats

**Laboratory:** Monsanto Agricultural Company, St. Louis, Missouri

**In-Life Dates:** 5 August, 1987 to 10 August, 1989

**Guidelines Used:** US-EPA Pesticide Assessment Guidelines Subdivision F, 83-5 (1982)

**Test material:** Glyphosate Technical

**Purity:** 96.5%

**Route of Exposure:** Diet

**Species:** Rat

**Strain:** Sprague-Dawley (CD) from Charles River Breeding Lab, Portage, MI

**Age at start:** Approximately 8 weeks

**Sex:** Males and Females

**Diet:** Purina Certified Rodent Chow #5002

**Housing:** Individual rats in stainless steel cages with wire mesh bottoms

**Dosing:** 0, 2,000, 8,000 and 20,000 ppm

**Animals per group (main study):** 50

**Duration of exposure (main study):** 24 months

**Interim Sacrifice:** 12 months

**Number of animals per group (interim sacrifice):** 10

**Tissues examined:** adrenals, aorta, bone & bone marrow, brain, caecum, colon, duodenum, eyes, gross lesions including palpable masses, Harderian gland, heart, ileum, jejunum, kidneys, liver, lung (with main stem bronchi), lymph nodes (mesenteric and submandibular), muscle, nasal turbinates, oesophagus, ovaries, pancreas, pituitary, prostrate, rectum, sciatic nerve, seminal vesicles, skin (with mammary tissue), spinal cord (cervical, thoracic, lumbar), spleen, stomach, submaxillary salivary gland, testes with epididymis, thymus, thyroid/parathyroid, trachea, urinary bladder, uterus (corpus and cervix).

**Pathology protocol:** A detailed histopathological examination was performed on all sampled tissues in all dose groups.

**Tumors evaluated in this reanalysis:**

**Males:** Adrenals: Pheochromocytoma benign, malignant, combined, Cortical adenoma; Brain: Astrocytoma; Kidney: Tubular adenoma; Liver: Hepatocellular adenoma, carcinoma, combined; Pancreas: Islet cell adenoma, carcinoma, combined, Reticulum cell sarcoma; Parathyroid Gland; Adenoma; Pituitary Gland: Adenoma (pars distalis); Skin: Keratoacanthoma, Basosquamous cell tumor, Squamous papilloma; Spleen: Reticulum cell sarcoma; Subcutis: Fibroma, Lipoma; Testes: Interstitial cell tumor; Thyroid Gland: C-cell adenoma, carcinoma, combined, Follicular cell adenoma, carcinoma, combined;

**Total number of trend tests: 29 (1 historical control)**

**Females:** Adrenals: Cortical adenoma, carcinoma, combined; Liver: Reticulum cell sarcoma, malignant lymphoma, Hepatocellular adenoma, carcinoma, combined; Lung: Reticulum cell sarcoma; Lymph Node, Mediastinal: Reticulum cell sarcoma, malignant lymphoma; Mammary Gland: Adenoma/Fibroadenoma/Fibroma, Adenocarcinoma, combined; Ovary: Granulosa cell tumor; Pancreas: Islet cell adenoma; Pituitary Gland: Adenoma (pars distalis); Thyroid Gland: C-cell adenoma, carcinoma, combined, Follicular cell, adenoma or carcinoma; Uterus: Polyp

**Total number of trend tests: 22**

*Atkinson et al. (1993) [19] – Study I*

**Title:** 104 week combined chronic feeding / oncogenicity study in rats with 52 week interim kill (results after 104 weeks)

**Laboratory:** Inveresk Research International Limited, Elphinstone Research Center, Tranent, Scotland

**In-Life Dates:** 16 February, 1990 to 9 March, 1992

**Guidelines Used:** US-EPA Pesticide Assessment Guidelines Subdivision F, 83-5 (1982)

**Test material:** Glyphosate Technical

**Purity:** 98.7% to 98.9%

**Route of Exposure:** Diet

**Species:** Rat

**Strain:** Sprague-Dawley Rats from Charles River (UK) Limited Margate, Kent, UK

**Age at start:** Approximately 6 weeks

**Sex:** Males and Females

**Diet:** Finely ground #1 Diet SQC Expanded from Special Diets Services Limited, Essex

**Housing:** Five animals per sex in polypropylene cages

**Dosing:** 0, 10, 100, 300 and 1,000 mg/kg/day

**Animals per group (main study):** 50

**Duration of exposure (main study):** 24 months

**Interim Sacrifice:** 52 weeks

**Number of animals per group (interim sacrifice):** 15

**Tissues examined:** adrenals, aortic arch, any abnormal tissue, bladder, bone and bone marrow (sternum and rib), brain, ears, eyes, intestine (duodenum, jejunum, ileum, caecum, colon, rectum), kidneys, liver, lungs, mammary gland, lymph nodes (mesenteric and submandibular), muscle (thigh), nasal cavity (oncogenicity study only), oesophagus, optic nerve, ovaries (with fallopian tubes), pancreas, parotid salivary glands, pituitary, prostrate, sciatic nerve, seminal vesicles, skin, spinal cord (cervical, thoracic and lumbar), spleen, stomach (glandular and non-glandular), sublingual salivary glands, submaxillary salivary glands, testes with epididymes, thymus, thyroid/parathyroid, tongue, trachea, uterus and vagina

**Pathology protocol:** Histopathological examination was carried out on all tissues collected from control and high dose animals, all animals that died or were killed in extremis during the study, all gross and palpable lesions, livers, lungs, and kidneys of all animals.

**Tumors evaluated in this reanalysis:**

**Males:** Adrenals: Pheochromocytoma benign, malignant, combined; Brain: Glioma; Kidney: Tubular adenoma; Liver: Hepatocellular adenoma, carcinoma, combined; Lung: Alveolar/Bronchiolar adenoma; Lymphoreticular tissue: Histiocytic Sarcoma, Malignant lymphoma; Pancreas: Exocrine adenoma, Islet cell adenoma, carcinoma, combined; Pituitary Gland: Adenoma; Skin: Cornifying epithelioma (keratoacanthoma), Basosquamous cell tumor, Fibrosarcoma, Fibroma; Testes: Interstitial cell tumor; Thyroid Gland: C-cell adenoma, carcinoma, combined, Follicular cell adenoma, carcinoma, combined;

**Total number of trend tests: 27**

**Females:** Adrenals: Cortical adenoma, carcinoma, combined; Liver: Reticulum cell sarcoma, malignant lymphoma, Hepatocellular adenoma; Lymphoreticular tissue: Histiocytic sarcoma, Lymphoma; Mammary Gland: Fibroadenoma, Adenoma, Carcinoma, combined; Pancreas: Islet cell adenoma; Pituitary Gland: Adenoma, carcinoma, combined; Thyroid Gland: C-cell adenoma, carcinoma, combined, Follicular cell, adenoma or carcinoma

**Total number of trend tests: 22**

*Enemoto (1997) [20] – Study J*

**Title:** 24-Month Oral Chronic Toxicity and Oncogenicity Study in Rats

**Laboratory:** The Institute of Environmental Toxicology, Tokyo, Japan

**In-Life Dates:** 21 February, 1995 to 6 September, 1996

**Guidelines Used:** Japan MAFF Guidelines 59 NohSan No.3850, 1984, U.S. EPA FIFRA Guidelines Subdivision F, 1989, OECD 451 (1981).

**Test material:** Glyphosate Technical

**Purity:** 94.61% to 97.56%

**Route of Exposure:** Diet

**Species:** Rat

**Strain:** Sprague-Dawley (Crj:CD) from Charles River Japan, Inc., Tsukuba Breeding Center

**Age at start:** 5 weeks (males), 6 weeks (females)

**Sex:** Males and Females

**Diet:** Certified diet MF Mash, Oriental Yeast Co., Ltd.

**Housing:** Males were housed 5 per cage until week 72, in groups of ≤3 until week 78 and individually thereafter. Females were housed in groups of five until week 78, and individually thereafter.

**Dosing:** 0, 3,000, 10,000 and 30,000 ppm

**Animals per group (main study):** 50

**Duration of exposure (main study):** 104 weeks

**Interim Sacrifice:** 26, 52 and 78 weeks

**Number of animals per group (interim sacrifice):** 10

**Tissues examined:** adrenals, aorta, bone & bone marrow (sternum and femur incl. joint), brain (cerebrum, cerebellum, pons and medulla oblongata), caecum, colon, duodenum, epididymides, eyes, gross lesions, Harderian glands, heart, ileum, jejunum, kidneys, liver, lungs, mammary gland, lymph nodes (cervical and mesenteric), oesophagus, ovaries, pancreas, pituitary, prostrate, rectum, salivary glands (submaxillary and sublingual), sciatic nerve, seminal vesicles and coagulating glands, skeletal muscle, skin (females only), spinal cord (cervical, thoracic and lumbar), spleen, stomach, testes, thymus, thyroid/parathyroid, trachea, urinary bladder, uterus (horns and cervix) and vagina.

**Pathology protocol:** A detailed histopathological examination was performed on all sampled tissues in all dose groups.

**Tumors evaluated in this reanalysis:**

**Males:** Adrenals: Cortical adenoma, Pheochromocytoma; Brain: Glioma; General: Malignant lymphoma; Heart: Schwannoma; Kidney: Tubular adenoma; Liver: Hepatocellular adenoma, carcinoma, combined; Lung: Alveolar/Bronchiolar adenoma, adenocarcinoma, combined; Mammary Gland: Fibroadenoma, Adenoma, Adenocarcinoma, combined; Pancreas: Acinar-cell adenoma, Islet cell adenoma, carcinoma, combined; Pituitary Gland: Adenoma; Skin: Papilloma, Keratoacanthoma, Basal cell tumor, Fibroma, Lipoma; Testes: Interstitial cell tumor; Thyroid Gland: C-cell adenoma, carcinoma, combined, Follicular cell adenoma, carcinoma, combined;

**Total number of trend tests: 33**

**Females:** Adrenals: Cortical adenoma, carcinoma, combined, Pheochromocytoma; General: Malignant lymphoma; Mammary Gland: Fibroadenoma, Adenonoma, Carcinoma, combined; Pancreas: Islet cell adenoma, carcinoma, combined; Pituitary Gland: Adenoma; Thyroid Gland: C-cell adenoma, carcinoma, combined, Follicular cell adenoma; Uterus: Polyp

**Total number of trend tests: 18**

*Suresh (1996) [21] – Study K*

**Title:** Combined Chronic Toxicity and Carcinogenicity Study with Glyphosate Technical in Wistar Rats

**Laboratory:** Rallis Research Centre, Peenya, Bangalore, India

**In-Life Dates:** 4 March, 1992 to 4 March, 1994

**Guidelines Used:** OECD 453 (1981)

**Test material:** Glyphosate Technical

**Purity:** 96.8%

**Route of Exposure:** Diet

**Species:** Rat

**Strain:** Wistar (in house colony)

**Age at start:** Six weeks

**Sex:** Males and Females

**Diet:** Standard "Gold Mohur" from M/S Lipton India Ltd, India

**Housing:** Initially in groups of five per sex in polypropylene cages and in groups of three from Week 12 onwards.

**Dosing:** 0, 100, 1,000 and 10,000 ppm

**Animals per group (main study):** 50

**Duration of exposure (main study):** 24 Months

**Interim Sacrifice:** 12 months

**Number of animals per group (interim sacrifice):** 10 per group except high dose with 20

**Tissues examined:** adrenals, aorta (main group animals), bone & bone marrow (sternum and femur incl. joint), brain, caecum, colon, duodenum, epididymides (main group animals), eyes (with optic nerve), heart, ileum, jejunum, kidneys, liver, lungs, mammary gland, lymph nodes (mesenteric, mandibular and mediastinal), muscle (femoral), oesophagus, ovaries, pancreas, pituitary, prostrate, rectum, salivary glands, sciatic nerve, seminal vesicles and coagulating glands, skin, spinal cord (cervical, thoracic and lumbar), spleen, stomach, testes, thymus, thyroid/parathyroid, trachea, tumour/mass, urinary bladder and uterus.

**Pathology protocol:** Histopathological examination was carried out on all tissues collected from control and high dose animals, all animals that died or were killed in extremis during the study, all gross and palpable lesions.

**Tumors evaluated in this reanalysis:**

**Males:** Adrenal Gland: Cortical cell adenoma, Pheochromocytoma benign, malignant, combined; Liver: Intrahepatic bile duct adenoma, Hepatocholangiocarcinoma, Histiocytic sarcoma, Tumor emboli, Hepatocellular adenoma, carcinoma, combined; Pancreas: Islet cell adenoma; Pituitary: Adenoma, Adenocarcinoma, combined; Skin-cutaneous: Keratoacanthoma; Testes: Leydig cell tumor; Thyroid Gland: C-cell adenoma

**Total number of trend tests: 18**

**Females:** Adrenals: Cortical cell adenoma, Pheochromocytoma; Liver: Hepatocellular adenoma, carcinoma, combined, Histiocytic sarcoma; Lung: Alveolar/bronchiolar adenoma; Mammary Gland: Adenoma, Adenocarcinoma, combined; Pituitary Gland: Adenoma, Adenocarcinoma, combined; Thyroid Gland: C-cell adenoma; Uterus: Adenocarcinoma;

**Total number of trend tests: 15**

*Brammer (2001) [22] – Study L*

**Title:** Glyphosate Acid: Two Year Dietary Toxicity and Oncogenicity Study in Rats

**Laboratory:** Central Toxicology Laboratory, Macclesfield, Cheshire, UK

**In-Life Dates:** 7 April, 1998 to 16 Ocyober, 2000

**Guidelines Used:** OECD 453 (1981)

**Test material:** Glyphosate Technical

**Purity:** 96.8%

**Route of Exposure:** Diet

**Species:** Rat

**Strain:** Wistar (Alpk:APfSD) from AstraZeneca UK Ltd, Macclesfield, Cheshire, UK

**Age at start:** Five to six weeks

**Sex:** Males and females

**Diet:** CT1 diet from Special Diets Services Ltd., Essex, UK

**Housing:** Four per sex per cage (cage type unknown)

**Dosing:** 0, 2,000, 6,000 and 20,000 ppm

**Animals per group (main study):** 53

**Duration of exposure (main study):** 103 weeks (males), 104 weeks (females)

**Interim Sacrifice:** 52 weeks

**Number of animals per group (interim sacrifice):** 12

**Tissues examined:** adrenals, aorta, bone & bone marrow (femur incl. joint), brain (cerebrum, cerebellum, brainstem), caecum, cervix, colon, duodenum, epididymis, eyes (retina, optic nerve), gross lesions including palpable masses, Harderian gland, heart, ileum, jejunum, kidneys, lachrymal gland, larynx, liver, lung, lymph nodes (cervical and mesenteric), mammary gland, muscle, oesophagus, ovary, pancreas, pharynx, pituitary, prostrate, rectum, salivary glands (submandibular, parotid), seminal vesicles, skin, spinal cord (cervical, thoracic, lumbar), spleen, sternum, stomach, testes, thymus, thyroid/parathyroid, trachea, urinary bladder and uterus.

**Pathology protocol:** A detailed histopathological examination was performed on all sampled tissues in all dose groups.

**Tumors evaluated in this reanalysis:**

**Males:** Adrenal Gland: Pheochromocytoma benign, malignant, combined; Liver: Hepatocellular adenoma, carcinoma, combined; Lymph Node, Mesenteric: Hemangioma; Lymphoreticular system: Large granular lymphocytic leukemia; Oral Cavity: Squamous cell carcinoma; Pancreas: Exocrine adenoma, Islet cell adenoma; Pituitary: Adenoma, Adenocarcinoma, combined; Skin-cutaneous: Basal cell tumor, Pilomatrixoma, Keratoacanthoma, Sebaceous adenoma; Subcutaneous tissue: Fibroma, Fibrosarcoma, Lipoma; Testes: Leydig cell tumor; Thymus: Thymoma; Thyroid Gland: Follicular cell adenoma, Parafollicular cell adenoma

**Total number of trend tests: 26 (2 historical controls)**

**Females:** Adrenals: Cortical cell adenoma; Brain: Astrocytoma; Cervix: Stromal cell sarcoma; Lymph Node, Mesenteric: Hemangioma; Lymphoreticular system: Large granular lymphocytic leukemia; Lung: Alveolar/bronchiolar adenoma; Mammary Gland: Mammary Gland: Fibroadenoma, Adenoma, Adenocarcinoma, Adenoma or Adenocarcinoma, Fibro/Adenoma or Adenocarcinoma, Cystadenoma; Oral Cavity: Squamous cell carcinoma; Pituitary Gland: Adenoma, Adenocarcinoma, combined; Subcutaneous tissue: Fibroma, Lipoma; Thyroid Gland: Follicular cell adenoma, Parafollicular cell adenoma; Uterus: Stromal cell polyp , Adenocarcinoma;

**Total number of trend tests: 22**

*Wood et al. (2009) [23] – Study M*

**Title:** Glyphosate Technical: Dietary Combined Chronic Toxicity/Carcinogenicity in the Rat

**Laboratory:** Harlan Laboratories Limited, Shardlow, UK

**In life dates:** 2005-09-01 to 2007-08-31

**Guidelines Used:** OECD 453 (1981), JMAFF Guideline 2-1-16 (2005), US OPTTS 870.4300 (1996)

**Test material:** Glyphosate Technical

**Purity:** 95.7 % w/w

**Route of Exposure:** Diet

**Species:** Rat

**Strain:** Wistar Ham Crl:WI (GLx/BRL/Han)IGS BR

**Age at start:** 5-6 weeks

**Sex:** Males and females

**Diet:** Unknown

**Housing:** Three per sex in polypropylene solid floor cages

**Dosing:** 0, 1,500, 5,000 and 15,000 ppm

**Animals per group (main study):** 51

**Duration of exposure (main study):** 24 months

**Interim Sacrifice:** 12 months

**Number of animals per group (interim sacrifice):** 15

**Tissues examined:** adrenals, aorta (thoraic), bone & bone marrow (sternum and femur incl. joint), brain (cerebrum, cerebellum, pons), caecum, colon, duodenum, epididymides, eyes (with optic nerve), gross lesions including palpable masses, head (pharynx, nasopharynx, paranasal sinuses), heart, Harderian gland, ileum (incl. Peyer's patches), jejunum, kidneys, liver, lungs (with bronchi), lymph nodes (cervical and mesenteric), mammary gland, muscle (skeletal), oesophagus, ovaries, pancreas, pituitary, prostrate, rectum, salivary glands (submaxillary), sciatic nerve, seminal vesicles, skin (hind limb), spinal cord (cervical, mid-thoracic and lumbar), spleen, stomach, testes, thymus, thyroid/parathyroid, tongue, trachea, urinary bladder, uterus and vagina.

**Pathology protocol:** Histopathological examination was carried out on all tissues collected from control and high dose animals, all animals that died or were killed in extremis during the study, all gross and palpable lesions, livers, lungs, kidneys and bone marrow of all animals.

**Tumors evaluated in this reanalysis:**

**Males:** Adrenal Gland: Pheochromocytoma benign, malignant, combined; Liver: Hepatocellular adenoma, carcinoma, combined; Lymph Node: Angioma, Angiosarcoma; Nasal cavities: Polypoid adenoma; Pancreas: Islet cell adenoma; Pituitary: Adenoma, Adenocarcinoma, combined; Skin-cutaneous: Squamous cell carcinoma, Keratoacanthoma; Skin-subcutaneous: Fibroma, Fibrosarcoma; Stomach: Squamous papilloma; Testes: Interstitial cell tumor; Thyroid Gland: Follicular adenoma, Parafollicular adenoma, Parafollicular adenocarcinoma, combined;

**Total number of trend tests: 23**

**Females:** Liver: Hepatocellular adenoma; Lymph Node: Angioma; Lymphoid/Hematopoetic: Malignant lymphoma; Mammary Gland: Fibroadenoma, Adenoma, Adenocarcinoma, Adenoma or Adenocarcinoma, Fibro/Adenoma or Adenocarcinoma; Ovary: Granulosa cell tumor, Granulosa theca cell tumor; Pituitary Gland: Adenoma, Adenocarcinoma, Adenoma or Adenocarcinoma; Thymus: Lymphocytic thymoma; Thyroid Gland: Follicular adenoma, adenocarcinoma, combined, Parafollicular adenoma, Uterus: Endometrial stromal polyp, Adenocarcinoma, Leiomyoma;

**Total number of trend tests: 21**
