# Supplementary material for: A comprehensive analysis of the animal carcinogenicity data for glyphosate from chronic exposure rodent carcinogenicity studies
Source: Environ Health. 2020 Feb 12;19:18. doi: 10.1186/s12940-020-00574-1 (PMC7014589; doi:10.1186/s12940-020-00574-1)
Supplement: Supplementary file 2 — Additional file 2: Table S1. Tumors of interest in male and female CD-1 mice from the 24-month feeding study of Knezevich and Hogan (1983) [11] – Study A. Table S2. Tumors of interest in male and female CD-1 mice from the 24-month feeding study of Atkinson et al. (1993) [12] – Study B. Table S3. Tumors of interest in male and female CD-1 mice from the 18-month feeding study of Sugimoto (1997) [13] – Study C. Table S4. Tumors of interest in male and female CD-1 mice from the 18-month feeding study of Wood et al. (2009) [14] – Study D. Table S5. Tumors of interest in male and female CD-1 mice from the 18-month feeding study of Takahashi (1999) [15]; data extracted from JMPR [7] – Study E. Table S6. Tumors of interest in male and female Swiss Albino mice from the 18-month feeding study of Kumar (2001) [16] – Study F. Table S7. Tumors of interest in male and female Sprague-Dawley rats the 26-month feeding study of Lankas (1981) [17] – Study G. Table S8. Tumors of interest in male and female Sprague-Dawley rats from the 24-month feeding study of Stout and Ruecker (1990) [18] – Study H. Table S9. Tumors of interest in male and female Sprague-Dawley rats from the 24-month feeding study of Atkinson et al. (1993) [19] – Study I. Table S10. Tumors of interest in male and female Sprague-Dawley rats from the 24-month feeding study of Enemoto (1997) [20] – Study J. Table S11. Tumors of interest in male and female Wistar rats from the 24-month feeding study of Suresh (1996) [21] – Study K. Table S12. Tumors of interest in male and female Wistar rats from the 24-month feeding study of Brammer (2001) [22] – Study L. Table S13. Tumors of interest in male and female Wistar rats from the 24-month feeding study of Wood et al. (2009) [23] – Study M. Table S14. Observed (Obs.) versus expected (Exp.) tumor sites with significant trends in the 13 acceptable rodent carcinogenicity studies using glyphosate. [file 12940_2020_574_MOESM2_ESM.docx]

**Additional file 2**

**Table S1.** Tumors of interest in male and female CD-1 mice from the 24-month feeding study of Knezevich and Hogan (1983) [11] – Study A

| Tumor | Doses (mg/kg/day) or Tumor Incidence^1^ | | | | Trend Test p-value |
| --- | --- | --- | --- | --- | --- |
| Males | 0 | 157 | 814 | 4841 |  |
| Kidney Adenomas (original pathology) | 0/49 | 0/49 | 1/50 | 3/50 | 0.019 |
| Kidney Adenomas^2^ | 1/49 | 0/49 | 0/50 | 1/50 | 0.442 |
| Kidney Carcinomas^2^ | 0/49 | 0/49 | 1/50 | 2/50 | 0.063 |
| Kidney Adenomas and Carcinomas^2^ | 1/49 | 0/49 | 1/50 | 3/50 | 0.065 |
| Malignant Lymphomas | 2/49 | 5/49 | 4/50 | 2/50 | 0.754 |
| Hemangiosarcomas^3^ | 0/49 | 0/49 | 1/50 | 0/50 | 0.505 |
| Alveolar-Bronchiolar Adenomas | 5/48 | 9/50 | 9/50 | 9/50 | 0.294 |
| Alveolar-Bronchiolar Carcinomas | 4/48 | 3/50 | 2/50 | 1/50 | 0.918 |
| Alveolar-Bronchiolar Adenomas and Carcinomas | 9/48 | 12/50 | 11/50 | 10/50 | 0.576 |
| Females | 0 | 190 | 955 | 5874 |  |
| Hemangiomas | 0/49 | 1/49 | 1/50 | 0/50 | 0.631 |
| Harderian Gland Adenomas | 2/45 | 0/48 | 1/49 | 0/44 | 0.877 |
| Harderian Gland Carcinomas | 0/45 | 0/48 | 0/49 | 0/44 | --- |
| Harderian Gland Adenomas and Carcinomas | 2/45 | 0/48 | 1/49 | 0/44 | 0.877 |
| Alveolar-Bronchiolar Adenomas | 10/49 | 9/50 | 10/49 | 1/50 | 0.999 |
| Alveolar-Bronchiolar Carcinomas | 1/49 | 3/50 | 4/49 | 4/50 | 0.183 |
| Alveolar-Bronchiolar Adenomas and Carcinomas | 11/49 | 12/50 | 14/49 | 5/50 | 0.985 |
| Spleen Composite Lymphosarcoma | 1/50 | 1/48 | 1/49 | 5/49 | 0.016 |
| Malignant Lymphomas | 5/49 | 6/49 | 6/49 | 10/49 | 0.070 |

1 – Doses are given in the rows marked “Males” and “Females”, tumor counts appear on the rows with the individual tumors; 2 – tumor counts obtained from EPA]; * 0.01<p≤0.05 for Fisher’s Exact Test; ** p≤0.01 for Fisher’s Exact Test

**Table S2.** Tumors of interest in male and female CD-1 mice from the 24-month feeding study of Atkinson et al. (1993) [12] – Study B

| Tumor | Doses (mg/kg/day) or Tumor Incidence^1^ | | | | Trend Test p-value |
| --- | --- | --- | --- | --- | --- |
| Males | 0 | 98 | 297 | 988 |  |
| Kidney Adenomas | 1/50 | 1/50 | 0/50 | 0/50 | 0.938 |
| Kidney Carcinomas | 1/50 | 1/50 | 0/50 | 0/50 | 0.938 |
| Kidney Adenomas and Carcinomas | 2/50 | 2/50 | 0/50 | 0/50 | 0.981 |
| Malignant Lymphomas | 4/50 | 2/50 | 1/50 | 6/50 | 0.087 |
| Hemangiosarcomas | 0/50 | 0/50 | 0/50 | 4/50 | 0.004 |
| Alveolar-Bronchiolar Adenomas | 12/50 | 15/50 | 12/50 | 16/50 | 0.231 |
| Alveolar-Bronchiolar Carcinomas | 10/50 | 7/50 | 8/50 | 9/50 | 0.456 |
| Alveolar-Bronchiolar Adenomas and Carcinomas | 22/50 | 22/50 | 20/50 | 25/50 | 0.231 |
| Females | 0 | 102 | 298 | 1000 |  |
| Hemangiomas | 0/50 | 0/50 | 0/50 | 0/50 | --- |
| Harderian Gland Adenomas^2^ | Not Examined | | | | --- |
| Harderian Gland Carcinomas^2^ | 0/50 | 0/50 | 0/50 | 0/50 | --- |
| Harderian Gland Adenomas and Carcinomas^3^ | 0/50 | 0/50 | 0/50 | 0/50 | --- |
| Alveolar-Bronchiolar Adenomas | 7/50 | 5/49 | 3/50 | 9/50 | 0.144 |
| Alveolar-Bronchiolar Carcinomas | 3/50 | 2/49 | 1/50 | 5/50 | 0.110 |
| Alveolar-Bronchiolar Adenomas and Carcinomas | 10/50 | 7/49 | 4/50 | 14/50 | 0.048 |
| Malignant Lymphomas | 14/50 | 12/50 | 9/50 | 13/50 | 0.484 |

1 – Doses are given in the rows marked “Males” and “Females”, tumor counts appear on the rows with the individual tumors; 2 – No tumors were listed for this tissue in females, but there were no animals listed as evaluated histopathologically in this tissue; * 0.01<p≤0.05 for Fisher’s Exact Test; ** p≤0.01 for Fisher’s Exact Test

**Table S3.** Tumors of interest in male and female CD-1 mice from the 18-month feeding study of Sugimoto (1997) [13] – Study C

| Tumor | Doses (mg/kg/day) or Tumor Incidence^1^ | | | | Trend Test p-value |
| --- | --- | --- | --- | --- | --- |
| Males | 0 | 165 | 838.1 | 4348 |  |
| Kidney Adenomas | 0/50 | 0/50 | 0/50 | 2/50 | 0.062 |
| Kidney Carcinomas | 0/50 | 0/50 | 0/50 | 0/50 | --- |
| Kidney Adenomas and Carcinomas | 0/50 | 0/50 | 0/50 | 2/50 | 0.062 |
| Malignant Lymphomas | 2/50 | 2/50 | 0/50 | 6/50 | 0.016 |
| Hemangiosarcomas | 0/50 | 0/50 | 0/50 | 2/50 | 0.062 |
| Alveolar-Bronchiolar Adenomas | 8/50 | 14/50 | 13/50 | 11/50 | 0.513 |
| Alveolar-Bronchiolar Carcinomas | 1/50 | 1/50 | 6/50 | 4/50 | 0.148 |
| Alveolar-Bronchiolar Adenomas and Carcinomas | 9/50 | 15/50 | 19/50* | 15/50 | 0.294 |
| Females | 0 | 153.2 | 786.8 | 4116 |  |
| Hemangiomas | 0/50 | 0/50 | 2/50 | 5/50* | 0.002 |
| Harderian Gland Adenomas | 1/50 | 3/50 | 0/50 | 5/50 | 0.040 |
| Harderian Gland Carcinomas | 0/50 | 0/50 | 0/50 | 0/50 | --- |
| Harderian Gland Adenomas and Carcinomas | 1/50 | 3/50 | 0/50 | 5/50 | 0.040 |
| Alveolar-Bronchiolar Adenomas | 8/50 | 5/50 | 12/50 | 5/50 | 0.800 |
| Alveolar-Bronchiolar Carcinomas | 1/50 | 2/50 | 3/50 | 1/50 | 0.623 |
| Alveolar-Bronchiolar Adenomas and Carcinomas | 9/50 | 7/50 | 15/50 | 6/50 | 0.842 |
| Malignant Lymphomas | 6/50 | 4/50 | 8/50 | 7/50 | 0.294 |

1 – Doses are given in the rows marked “Males” and “Females”, tumor counts appear on the rows with the individual tumors; * 0.01<p≤0.05 for Fisher’s Exact Test; ** p≤0.01 for Fisher’s Exact Test

**Table S4.** Tumors of interest in male and female CD-1 mice from the 18-month feeding study of Wood et al. (2009) [14] – Study D

| Tumor | Doses (mg/kg/day) or Tumor Incidence^1^ | | | | Trend Test p-value |
| --- | --- | --- | --- | --- | --- |
| Males | 0 | 71.4 | 234.2 | 810 |  |
| Kidney Adenomas | 0/51 | 0/51 | 0/51 | 0/51 | --- |
| Kidney Carcinomas | 0/51 | 0/51 | 0/51 | 0/51 | --- |
| Kidney Adenomas and Carcinomas | 0/51 | 0/51 | 0/51 | 0/51 | --- |
| Malignant Lymphomas | 0/51 | 1/51 | 2/51 | 5/51* | 0.007 |
| Hemangiosarcomas | 0/51 | 0/51 | 0/51 | 0/51 | --- |
| Alveolar-Bronchiolar Adenomas | 9/51 | 7/51 | 9/51 | 4/51 | 0.924 |
| Alveolar-Bronchiolar Carcinomas | 5/51 | 5/51 | 7/51 | 11/51 | 0.028 |
| Alveolar-Bronchiolar Adenomas and Carcinomas | 14/51 | 12/51 | 16/51 | 15/51 | 0.336 |
| Females | 0 | 97.9 | 299.5 | 1081.2 |  |
| Hemangiomas | 0/51 | 2/51 | 0/51 | 1/51 | 0.438 |
| Harderian Gland Adenomas | 1/51 | 0/51 | 0/51 | 2/51 | 0.155 |
| Harderian Gland Carcinomas | 2/51 | 0/51 | 0/51 | 0/51 | 1.000 |
| Harderian Gland Adenomas and Carcinomas | 3/51 | 0/51 | 0/51 | 2/51 | 0.372 |
| Alveolar-Bronchiolar Adenomas | 2/51 | 4/51 | 2/51 | 2/51 | 0.656 |
| Alveolar-Bronchiolar Carcinomas | 5/51 | 2/51 | 2/51 | 3/51 | 0.601 |
| Alveolar-Bronchiolar Adenomas and Carcinomas | 7/51 | 6/51 | 4/51 | 5/51 | 0.688 |
| Malignant Lymphomas | 11/51 | 8/51 | 10/51 | 11/51 | 0.353 |

1 – Doses are given in the rows marked “Males” and “Females”, tumor counts appear on the rows with the individual tumors; * 0.01<p≤0.05 for Fisher’s Exact Test; ** p≤0.01 for Fisher’s Exact Test

**Table S5.** Tumors of interest in male and female CD-1 mice from the 18-month feeding study of Takahashi (1999) [15]; data extracted from JMPR [7] – Study E

| Tumor | Doses (mg/kg/day) or Tumor Incidence^1^ | | | | Trend Test p-value |
| --- | --- | --- | --- | --- | --- |
| Males | 0 | 167.6 | 685 | 7470 |  |
| Kidney Adenomas | 0/50 | 0/50 | 1/50 | 3/50 | 0.019 |
| Kidney Carcinomas | 0/50 | 0/50 | 0/50 | 1/50 | 0.250 |
| Kidney Adenomas and Carcinomas | 0/50 | 0/50 | 1/50 | 4/50 | 0.005 |
| Females | 0 | 93.2 | 909 | 8690 |  |
| Malignant Lymphomas | 3/50 | 1/50 | 4/50 | 6/50 | 0.050 |

1 – Doses are given in the rows marked “Males” and “Females”, tumor counts appear on the rows with the individual tumors; * 0.01<p≤0.05 for Fisher’s Exact Test; ** p≤0.01 for Fisher’s Exact Test

**Table S6.** Tumors of interest in male and female Swiss Albino mice from the 18-month feeding study of Kumar (2001) [16] – Study F

| Tumor | Doses (mg/kg/day) or Tumor Incidence^1^ | | | | Trend Test p-value |
| --- | --- | --- | --- | --- | --- |
| Males | 0 | 14.5 | 149.7 | 1453 |  |
| Kidney Adenomas | 0/50 | 0/26 | 1/26 | 2/50 | 0.090 |
| Kidney Carcinomas | 0/50 | 0/26 | 0/26 | 0/50 | ---- |
| Kidney Adenomas and Carcinomas | 0/50 | 0/26 | 1/26 | 2/50 | 0.090 |
| Malignant Lymphomas | 10/50 | 15/50 | 16/50 | 19/50* | 0.064 |
| Females | 0 | 15 | 151.2 | 1466.8 |  |
| Malignant Lymphomas | 18/50 | 20/50 | 19/50 | 25/50 | 0.070 |
| Hemangioma | 1/50 | 0/50 | 0/50 | 5/50 | 0.004 |

1 – Doses are given in the rows marked “Males” and “Females”, tumor counts appear on the rows with the individual tumors; * 0.01<p≤0.05 for Fisher’s Exact Test; ** p≤0.01 for Fisher’s Exact Test

**Table S7.** Tumors of interest in male and female Sprague-Dawley rats the 26-month feeding study of Lankas (1981) [17] – Study G

| Tumor | Doses (mg/kg/day) or Tumor Incidence^1^ | | | | Trend Test p-value |
| --- | --- | --- | --- | --- | --- |
| Males | 0 | 3.05 | 10.30 | 31.49 |  |
| Testicular Interstitial Cell Tumors | 0/50 | 3/50 | 1/50 | 6/50* | 0.009 |
| Pancreas Islet Cell Adenomas | 0/50 | 5/49* | 2/50 | 2/50 | 0.512 |
| Pancreas Islet Cell Carcinomas | 0/50 | 0/49 | 0/50 | 1/50 | 0.251 |
| Pancreas Islet Cell Adenomas or Carcinomas | 0/50 | 5/50* | 2/50 | 3/50 | 0.316 |
| Thyroid C-Cell Adenomas | 5/47 | 1/49 | 0/49 | 2/49 | 0.743 |
| Thyroid C-Cell Carcinomas | 0/47 | 0/49 | 1/49 | 0/49 | 0.505 |
| Thyroid C-Cell Adenomas and Carcinomas | 5/47 | 1/49 | 1/49 | 2/49 | 0.748 |
| Thyroid Follicular-Cell Adenomas | 1/47 | 2/49 | 4/49 | 4/49 | 0.122 |
| Thyroid Follicular-Cell Carcinomas | 0/47 | 0/49 | 0/49 | 0/49 | --- |
| Thyroid Follicular-Cell Adenomas and Carcinomas | 1/47 | 2/49 | 4/49 | 4/49 | 0.122 |
| Liver Neoplastic Nodules | 3/50 | 3/50 | 1/50 | 3/50 | 0.471 |
| Liver Carcinomas | 0/50 | 0/50 | 1/50 | 2/50 | 0.062 |
| Liver Nodules and Carcinomas | 3/50 | 3/50 | 2/50 | 5/50 | 0.173 |
| Kidney Adenomas | 1/50 | 1/50 | 0/50 | 0/50 | 0.938 |
| Skin Keratoacanthomas | 0/49 | 0/48 | 0/49 | 0/49 | --- |
| Skin Basal Cell Tumors | 0/49 | 0/48 | 0/49 | 1/49 | 0.251 |
| Females | 0 | 3.37 | 11.22 | 34.02 |  |
| Thyroid C-Cell Adenomas | 5/47 | 3/49 | 6/50 | 3/47 | 0.679 |
| Thyroid C-Cell Carcinomas | 1/47 | 0/49 | 2/50 | 6/47 | 0.003 |
| Thyroid C-Cell Adenomas and Carcinomas | 6/47 | 3/49 | 8/50 | 9/47 | 0.072 |
| Adrenal Cortical Adenomas | 5/50 | 10/50 | 6/50 | 4/49 | 0.851 |
| Adrenal Cortical Carcinomas | 1/50 | 0/50 | 1/50 | 1/49 | 0.386 |
| Adrenal Cortical Adenomas and Carcinomas | 6/50 | 10/50 | 7/50 | 5/49 | 0.801 |

1 – Doses are given in the rows marked “Males” and “Females”, tumor counts appear on the rows with the individual tumors; * 0.01<p≤0.05 for Fisher’s Exact Test; ** p≤0.01 for Fisher’s Exact Test

**Table S8.** Tumors of interest in male and female Sprague-Dawley rats from the 24-month feeding study of Stout and Ruecker (1990) [18] – Study H

| Tumor | Doses (mg/kg/day) or Tumor Incidence^1^ | | | | Trend Test p-value |
| --- | --- | --- | --- | --- | --- |
| Males | 0 | 89 | 362 | 940 |  |
| Testicular Interstitial Cell Tumors | 2/50 | 0/50 | 3/50 | 2/50 | 0.296 |
| Pancreas Islet Cell Adenomas | 1/48 | 8/47* | 5/50 | 7/49* | 0.147 |
| Pancreas Islet Cell Carcinomas | 1/48 | 0/47 | 0/50 | 0/49 | 1.000 |
| Pancreas Islet Cell Adenomas or Carcinomas | 2/48 | 8/47* | 5/50 | 7/49* | 0.206 |
| Thyroid C-cell Adenomas | 0/50 | 4/48 | 8/48** | 5/50* | 0.089 |
| Thyroid C-cell Carcinomas | 0/50 | 2/48 | 0/48 | 1/50 | 0.442 |
| Thyroid C-cell Adenomas and Carcinomas | 0/50 | 6/50* | 8/50** | 6/50* | 0.097 |
| Thyroid Follicular-cell Adenomas | 2/50 | 1/48 | 3/48 | 2/50 | 0.408 |
| Thyroid Follicular-cell Carcinomas | 0/50 | 0/48 | 0/48 | 1/50 | 0.255 |
| Thyroid Follicular-cell Adenoma and Carcinoma | 2/50 | 1/48 | 3/48 | 3/50 | 0.232 |
| Hepatocellular Adenomas | 3/50 | 2/50 | 3/50 | 8/50 | 0.015 |
| Hepatocellular Carcinomas | 3/50 | 2/50 | 1/50 | 2/50 | 0.637 |
| Hepatocellular Adenomas and Carcinomas | 6/50 | 4/50 | 4/50 | 10/50 | 0.050 |
| Kidney Adenomas | 0/50 | 2/50 | 0/50 | 0/50 | 0.813 |
| Skin Keratoacanthomas | 0/49 | 3/46 | 4/50 | 5/48 | 0.042 |
| Skin Basal Cell Tumors | 0/49 | 0/46 | 0/50 | 1/48 | 0.249 |
| Females | 0 | 113 | 457 | 1183 |  |
| Thyroid C-cell Adenomas | 2/50 | 2/50 | 6/50 | 6/50 | 0.049 |
| Thyroid C-cell Carcinomas | 0/50 | 0/50 | 1/50 | 0/50 | 0.500 |
| Thyroid C-cell Adenomas and Carcinomas | 2/50 | 2/50 | 7/50 | 6/50 | 0.052 |
| Adrenal Cortical Adenoma | 1/50 | 2/50 | 2/50 | 1/50 | 0.603 |
| Adrenal Cortical Carcinoma | 0/50 | 0/50 | 0/50 | 3/50 | 0.015 |
| Adrenal Cortical Adenoma and Carcinoma | 1/50 | 2/50 | 2/50 | 4/50 | 0.090 |

1 – Doses are given in the rows marked “Males” and “Females”, tumor counts appear on the rows with the individual tumors; * 0.01<p≤0.05 for Fisher’s Exact Test; ** p≤0.01 for Fisher’s Exact Test

**Table S9**. Tumors of interest in male and female Sprague-Dawley rats from the 24-month feeding study of Atkinson et al. (1993) [19] – Study I

| Tumor | Doses (mg/kg/day) or Tumor Incidence^1^ | | | | | Trend Test p-value |
| --- | --- | --- | --- | --- | --- | --- |
| Males | 0 | 11 | 112 | 320 | 1147 |  |
| Testicular Interstitial Cell Tumors | 3/50 | 1/25 | 0/19 | 0/21 | 2/50 | 0.580 |
| Pancreas Islet Cell Adenomas | 7/50 | 1/24 | 2/17 | 2/21 | 1/49 | 0.974 |
| Pancreas Islet Cell Carcinomas | 0/50 | 0/24 | 0/17 | 0/21 | 0/49 | --- |
| Pancreas Islet Cell Adenomas or Carcinomas | 7/50 | 1/24 | 2/17 | 2/21 | 1/49 | 0.974 |
| Thyroid C-cell Adenomas | 9/50 | 1/21 | 1/17 | 2/21 | 8/49 | 0.278 |
| Thyroid C-cell Carcinomas | 0/50 | 0/21 | 0/17 | 1/21 | 1/49 | 0.178 |
| Thyroid C-cell Adenomas and Carcinomas | 9/50 | 1/21 | 1/17 | 2/21 | 9/49 | 0.197 |
| Thyroid Follicular-cell Adenomas | 0/50 | 0/21 | 0/17 | 1/21 | 2/49 | 0.067 |
| Thyroid Follicular-cell Carcinomas | 0/50 | 0/21 | 0/17 | 1/21 | 0/49 | 0.443 |
| Thyroid Follicular-cell Adenoma and Carcinoma | 0/50 | 0/21 | 0/17 | 2/21 | 2/49 | 0.099 |
| Hepatocellular Adenomas | 2/50 | 1/50 | 1/49 | 2/50 | 2/50 | 0.325 |
| Hepatocellular Carcinomas | 0/50 | 1/50 | 1/49 | 0/50 | 0/50 | 0.760 |
| Hepatocellular Adenomas and Carcinomas | 2/50 | 2/50 | 2/49 | 2/50 | 2/50 | 0.480 |
| Kidney Adenomas | 1/50 | 0/50 | 0/50 | 0/50 | 0/50 | 1.000 |
| Skin Epithelioma (Keratoacanthomas) | 1/50 | 2/25 | 0/19 | 0/21 | 5/50 | 0.047 |
| Skin Basal Cell Tumors | 1/50 | 0/25 | 0/19 | 0/21 | 0/50 | 1.000 |
| Females | 0 | 12 | 109 | 347 | 1134 |  |
| Thyroid C-cell Adenomas | 8/50 | 1/27 | 1/29 | 2/29 | 7/49 | 0.207 |
| Thyroid C-cell Carcinomas | 0/50 | 0/27 | 0/29 | 0/29 | 0/49 | --- |
| Thyroid C-cell Adenomas and Carcinomas | 8/50 | 1/27 | 1/29 | 2/29 | 7/49 | 0.207 |
| Adrenal Cortical Adenoma | 0/48 | 0/26 | 0/29 | 0/30 | 0/49 | --- |
| Adrenal Cortical Carcinoma | 0/48 | 0/26 | 0/29 | 2/30 | 0/49 | 0.493 |
| Adrenal Cortical Adenoma and Carcinoma | 0/48 | 0/26 | 0/29 | 2/30 | 0/49 | 0.493 |

1 – Doses are given in the rows marked “Males” and “Females”, tumor counts appear on the rows with the individual tumors; * 0.01<p≤0.05 for Fisher’s Exact Test; ** p≤0.01 for Fisher’s Exact Test

**Table S10.** Tumors of interest in male and female Sprague-Dawley rats from the 24-month feeding study of Enemoto (1997) [20] – Study J

| Tumor | Doses (mg/kg/day) or Tumor Incidence^1^ | | | | Trend Test p-value |
| --- | --- | --- | --- | --- | --- |
| Males | 0 | 104 | 354 | 1127 |  |
| Testicular Interstitial Cell Tumors | 3/49 | 2/50 | 0/50 | 2/50 | 0.594 |
| Pancreas Islet Cell Adenomas | 4/50 | 1/50 | 1/50 | 1/50 | 0.859 |
| Pancreas Islet Cell Carcinomas | 0/50 | 0/50 | 1/50 | 0/50 | 0.500 |
| Pancreas Islet Cell Adenomas or Carcinomas | 4/50 | 1/50 | 2/50 | 1/50 | 0.844 |
| Thyroid C-cell Adenomas | 4/50 | 9/49 | 2/49 | 5/50 | 0.631 |
| Thyroid C-cell Carcinomas | 2/50 | 0/49 | 1/49 | 1/50 | 0.565 |
| Thyroid C-cell Adenomas and Carcinomas | 6/50 | 9/49 | 3/49 | 6/50 | 0.642 |
| Thyroid Follicular-cell Adenomas | 3/50 | 1/49 | 1/49 | 0/50 | 0.966 |
| Thyroid Follicular-cell Carcinomas | 1/50 | 0/49 | 0/49 | 0/50 | 1.000 |
| Thyroid Follicular-cell Adenoma and Carcinoma | 4/50 | 1/49 | 1/49 | 0/50 | 0.986 |
| Hepatocellular Adenomas | 0/50 | 0/50 | 1/50 | 0/50 | 0.500 |
| Hepatocellular Carcinomas | 0/50 | 1/50 | 2/50 | 0/50 | 0.642 |
| Hepatocellular Adenomas and Carcinomas | 0/50 | 1/50 | 3/50 | 0/50 | 0.690 |
| Kidney Adenomas | 0/50 | 0/50 | 0/50 | 4/50 | 0.004 |
| Skin Keratoacanthomas | 3/50 | 3/50 | 0/50 | 7/50 | 0.029 |
| Skin Basal Cell Tumors | 0/50 | 0/50 | 0/50 | 4/50 | 0.004 |
| Females | 0 | 115 | 393 | 1247 |  |
| Thyroid C-cell Adenomas | 4/50 | 7/49 | 7/49 | 2/50 | 0.912 |
| Thyroid C-cell Carcinomas | 0/50 | 0/49 | 0/49 | 0/50 | --- |
| Thyroid C-cell Adenomas and Carcinomas | 4/50 | 7/49 | 7/49 | 2/50 | 0.912 |
| Adrenal Cortical Adenoma | 0/50 | 1/50 | 1/50 | 0/50 | 0.626 |
| Adrenal Cortical Carcinoma | 0/50 | 0/50 | 0/50 | 0/50 | --- |
| Adrenal Cortical Adenoma and Carcinoma | 0/50 | 1/50 | 1/50 | 0/50 | 0.626 |

1 – Doses are given in the rows marked “Males” and “Females”, tumor counts appear on the rows with the individual tumors; * 0.01<p≤0.05 for Fisher’s Exact Test; ** p≤0.01 for Fisher’s Exact Test

**Table S11.** Tumors of interest in male and female Wistar rats from the 24-month feeding study of Suresh (1996) [21] – Study K

| Tumor | Doses (mg/kg/day) or Tumor Incidence^1^ | | | | Trend Test p-value |
| --- | --- | --- | --- | --- | --- |
| Males | 0 | 6.3 | 59.4 | 595.2 |  |
| Hepatocellular Adenomas | 24/50 | 22/50 | 10/48 | 21/50 | 0.391 |
| Hepatocellular Carcinomas | 21/50 | 28/50 | 18/48 | 24/50 | 0.418 |
| Hepatocellular Adenomas and Carcinomas | 45/50 | 50/50 | 28/48 | 45/50 | 0.286 |
| Pituitary Adenomas | 3/49 | 4/30 | 3/31 | 5/49 | 0.376 |
| Pituitary Carcinomas | 0/49 | 1/30 | 0/31 | 0/49 | 0.692 |
| Pituitary Adenomas and Carcinomas | 3/49 | 5/30 | 3/31 | 5/49 | 0.454 |
| Skin Keratoacanthomas | 0/50 | 0/30 | 0/32 | 0/50 | --- |
| Adrenal Pheochromocytomas | 13/50 | 5/33 | 7/36 | 17/50 | 0.048 |
| Females | 0 | 8.6 | 88.5 | 886 |  |
| Mammary Gland Adenomas | 2/40 | 3/30 | 8/33* | 5/48 | 0.539 |
| Mammary Gland Adenocarcinomas | 3/40 | 0/30 | 0/33 | 0/48 | 1.000 |
| Mammary Gland Adenomas and Adenocarcinomas | 5/40 | 3/30 | 8/33 | 5/48 | 0.729 |
| Pituitary Adenomas | 7/49 | 13/33 | 7/23 | 6/50 | 0.967 |
| Pituitary Carcinomas | 1/49 | 0/33 | 0/23 | 0/50 | 1.000 |
| Pituitary Adenomas and Carcinomas | 8/49 | 13/33 | 7/23 | 6/50 | 0.976 |

1 – Doses are given in the rows marked “Males” and “Females”, tumor counts appear on the rows with the individual tumors; * 0.01<p≤0.05 for Fisher’s Exact Test; ** p≤0.01 for Fisher’s Exact Test

**Table S12.** Tumors of interest in male and female Wistar rats from the 24-month feeding study of Brammer (2001) [22] – Study L

| Tumor | Doses (mg/kg/day) or Tumor Incidence^1^ | | | | Trend Test p-value |
| --- | --- | --- | --- | --- | --- |
| Males | 0 | 121 | 361 | 1214 |  |
| Hepatocellular Adenomas | 0/53 | 2/51 | 0/53 | 5/52* | 0.008 |
| Hepatocellular Carcinomas | 0/53 | 0/51 | 0/53 | 0/52 | --- |
| Hepatocellular Adenomas and Carcinomas | 0/53 | 2/51 | 0/53 | 5/52* | 0.008 |
| Pituitary Adenomas | 16/53 | 15/52 | 18/53 | 18/52 | 0.277 |
| Pituitary Carcinomas | 0/53 | 0/52 | 0/53 | 0/52 | --- |
| Pituitary Adenomas and Carcinomas | 16/53 | 15/52 | 18/53 | 18/52 | 0.277 |
| Skin Keratoacanthomas | 1/53 | 0/53 | 1/53 | 1/52 | 0.387 |
| Adrenal Pheochromocytomas | 8/53 | 5/53 | 7/52 | 5/52 | 0.721 |
| Females | 0 | 145 | 437 | 1498 |  |
| Mammary Gland Adenomas | 1/51 | 2/51 | 0/52 | 0/53 | 0.941 |
| Mammary Gland Adenocarcinomas | 2/51 | 0/51 | 0/52 | 2/53 | 0.271 |
| Mammary Gland Adenomas and Adenocarcinomas | 3/51 | 2/51 | 0/52 | 2/53 | 0.590 |
| Pituitary Adenomas | 42/51 | 40/51 | 42/52 | 45/53 | 0.261 |
| Pituitary Carcinomas | 0/51 | 0/51 | 0/52 | 0/53 | --- |
| Pituitary Adenomas and Carcinomas | 42/51 | 40/51 | 42/52 | 45/53 | 0.261 |

1 – Doses are given in the rows marked “Males” and “Females”, tumor counts appear on the rows with the individual tumors; * 0.01<p≤0.05 for Fisher’s Exact Test; ** p≤0.01 for Fisher’s Exact Test

**Table S13.** Tumors of interest in male and female Wistar rats from the 24-month feeding study of Wood et al. (2009) [23] – Study M

| Tumor | Doses (mg/kg/day) or Tumor Incidence^1^ | | | | Trend Test p-value |
| --- | --- | --- | --- | --- | --- |
| Males | 0 | 85.5 | 285.2 | 1077.4 |  |
| Hepatocellular Adenomas | 0/51 | 2/51 | 1/51 | 1/51 | 0.418 |
| Hepatocellular Carcinomas | 1/51 | 0/51 | 0/51 | 0/51 | 1.000 |
| Hepatocellular Adenomas and Carcinomas | 1/50 | 2/51 | 1/51 | 1/51 | 0.610 |
| Pituitary Adenomas | 16/51 | 11/51 | 10/51 | 20/51 | 0.045 |
| Pituitary Carcinomas | 1/50 | 0/51 | 0/51 | 0/51 | 1.000 |
| Pituitary Adenomas and Carcinomas | 17/51 | 11/51 | 10/51 | 20/51 | 0.059 |
| Skin Keratoacanthomas | 2/51 | 3/51 | 0/51 | 6/51 | 0.030 |
| Adrenal Pheochromocytomas | 2/51 | 0/51 | 3/51 | 2/51 | 0.306 |
| Females | 0 | 104.5 | 348.6 | 1381.9 |  |
| Mammary Gland Adenomas | 0/51 | 0/51 | 0/51 | 2/51 | 0.062 |
| Mammary Gland Adenocarcinomas | 2/51 | 3/51 | 1/51 | 6/51 | 0.042 |
| Mammary Gland Adenomas and Adenocarcinomas | 2/51 | 3/51 | 1/51 | 8/51* | 0.007 |
| Pituitary Adenomas | 24/51 | 23/51 | 16/51 | 32/51 | 0.014 |
| Pituitary Carcinomas | 0/51 | 1/51 | 0/51 | 0/51 | 0.750 |
| Pituitary Adenomas and Carcinomas | 24/51 | 24/51 | 16/51 | 32/51 | 0.017 |

1 – Doses are given in the rows marked “Males” and “Females”, tumor counts appear on the rows with the individual tumors; * 0.01<p≤0.05 for Fisher’s Exact Test; ** p≤0.01 for Fisher’s Exact Test

**Table S14.** Observed (Obs.) versus expected (Exp.) tumor sites with significant trends in the 13 acceptable rodent carcinogenicity studies using glyphosate

| Species | Strain | Sex | Total Sites^1^ | Exp. p<0.05 | Obs. p<0.05  (prob.)^2^ | Exp. p<0.01 | Obs. p<0.01 |
| --- | --- | --- | --- | --- | --- | --- | --- |
| Rat  (7 studies) | Sprague-Dawley  (4 studies) | M | 125 | 6.3 | 9 (0.17) | 1.3 | 4 (0.04) |
|  |  | F | 95 | 4.8 | 4 (0.52) | 1.0 | 2 (0.25) |
|  | Wistar  (3 studies) | M | 67 | 3.4 | 5 (0.24) | 0.7 | 2 (0.15) |
|  |  | F | 58 | 2.9 | 4 (0.33) | 0.6 | 1 (0.44) |
| Mouse  (6 studies) | CD-1  (5 studies) | M | 60 | 3.0 | 11 (<0.001) | 0.6 | 8 (<0.001) |
|  |  | F | 63 | 3.2 | 6 (0.09) | 0.6 | 1 (0.47) |
|  | Albino  (1 study) | M | 24 | 0.7 | 0 (1) | 0.1 | 0 (1) |
|  |  | F | 14 | 0.7 | 1 (0.51) | 0.1 | 1 (0.13) |
| Rats  (7 studies) | All  (7 studies) | M | 192 | 9.6 | 14 (0.10) | 1.9 | 6 (0.013) |
|  |  | F | 153 | 7.7 | 9 (0.36) | 1.5 | 3 (0.20) |
|  |  | Both | 345 | 17.3 | 23 (0.02) | 3.5 | 9 (0.01) |
| Mice  (6 studies) | All  (6 studies) | M | 74 | 3.7 | 11 (0.001) | 0.7 | 8 (<0.001) |
|  |  | F | 77 | 3.9 | 7 (0.09) | 0.8 | 2 (0.18) |
|  |  | Both | 151 | 7.6 | 18 (0.001) | 1.5 | 10 (<0.001) |
| All  (13 studies) | All  (13 studies) | M | 266 | 13.3 | 25 (0.002) | 2.7 | 14 (<0.001) |
|  |  | F | 230 | 11.5 | 16 (0.12) | 2.3 | 5 (0.08) |
|  |  | Both | 496 | 24.8 | 41 (0.001) | 5.0 | 19 (<0.001) |

1 – number of trend tests actually conducted; 2 – probability of seeing the number of observed significant findings or more
